# On the Utility of Integrated Speed-Accuracy Measures when Speed-Accuracy Trade-off is Present

**DOI:** 10.5334/joc.154

**Published:** 2021-03-18

**Authors:** André Vandierendonck

**Affiliations:** 1 Department of Experimental Psychology, Ghent University, Henri Dunantlaan 2, B-9000 Gent, Belgium

**Keywords:** Integrated Speed-Accuracy Measures, Speed-Accuracy Trade-Off, Drift-Diffusion Model

## Abstract

In an attempt to simplify data analysis and to avoid confounds due to speed-accuracy trade-off, sometimes integrated measures of speed and accuracy are used. Although it has been claimed that some of these combined measures are insensitive to speed-accuracy trade-off (SAT), a systematic and broad examination of such claims has not been performed thus far. The present article reports the results of four simulation studies in which five established integrated measures were studied in different speed-accuracy trade-off contexts. All four studies used repeated measures designs crossing an experimental factor (variable of interest) with a factor representing SAT settings, with all conditions occurring randomly over the sequence of trials to avoid condition-wise SATs (mixed conditions repeated measures design). The first study used speed modulations that were balanced by accuracy changes in the opposite direction. The other studies were all based on SAT as modeled either by the drift-diffusion model, with pro-active trade-off settings (Study 2) or with reactive trade-off modulations (Study 3) or by a discontinuous two-phase model (Study 4). Only the studies based on balanced trade-offs showed that two of the measures were insensitive to SAT settings, while in all other contexts, all measures were sensitive to SAT. Nevertheless, as the mixed conditions design distributes the SAT effects over the conditions of the variable of interest, all integrated measures reliably detected the effect of this variable in all SAT conditions. Although integrated measures are sensitive to SAT, these effects can be neutralised by using a mixed conditions repeated measures design.

Usage of measures that integrate speed and accuracy has been increasing in recent years. As only one measure instead of two is required, this has the advantage of simplifying performance reports in addition to achieving clarification whenever effects that are expected to be present in both speed and accuracy are not completely consistent. These measures are often used with the hope that when accuracy is traded for speed (or vice versa) the changes in accuracy (speed) will be compensated by the changes in speed (accuracy) so as to avoid or to neutralise speed-accuracy trade-offs. For some of these measures it has been claimed that they are insensitive to speed-accuracy trade-offs (Liesefeld & Janczyk, 2019) while it has also been suggested that they are not (Stafford, Pirrone, Croucher & Krystalli, 2020). The present article reports a set of simulation studies to investigate if and under which conditions it may be justifiable to use integrated speed-accuracy measures for the purpose of avoiding confounds due to speed-accuracy trade-off.

Speed (typically measured by response time, RT) and accuracy (typically measured by proportion of errors, PE, or its converse proportion correct responses, PC) are indeed two fundamental properties of performance in many tasks. Interestingly, when focusing on *task difficulty*, very often, though not always, the two measures are *positively correlated*, such that fast (low RT) and accurate (low PE) task performance is considered to correspond to an easy task, whereas slow (high RT) and error-prone (high PE) performance is associated with a difficult task. Under *speed-accuracy trade-off* (henceforth, SAT), in contrast, the two measures are *negatively correlated*. We all know from experience that speeded execution of an action increases the likelihood of committing an error and that higher accuracy can be achieved by a slower (i.e., more careful) task execution. Therefore, higher speed (lower RT) can be achieved by allowing more errors (higher PE) and higher accuracy (lower PE) can be obtained by slowing down responding (higher RT). In other words, when SAT is present, variations in speed and accuracy are negatively correlated (for reviews on SAT, see Heitz, 2014; Wickelgren, 1977).

Measures that integrate speed and accuracy in one single measure do so by combining the two aspects so that better performance on the integrated measure can be the result of higher speed or higher accuracy or both. Hence, if speed and accuracy correlate negatively in a particular task setting, it seems plausible to assume that the higher score contributed by one task property (e.g., speed) is—to some extent—compensated by a lower score contributed by the other property (e.g., accuracy). It is quite likely that these alleged compensation is partial, and whether the assumption of complete compensation is warranted depends on the specificity of the integrated measure under consideration and on the mechanisms that underly SAT.

## Integrated Speed-Accuracy Measures

First, the integrated speed-accuracy measures that form the focus of the present project are explained in more detail and attention is given to how they may neutralise SAT. A recent paper used simulations to compare a number of variants of different integrated measures that had been proposed thus far (Vandierendonck, 2017) and concluded that only three of the seven variants in the study were efficient to detect effects present in either RT or PE or both. More recently, an additional integrated measure has been proposed (Liesefeld & Janczyk, 2019). These measures are used in the simulations reported in the present article, and therefore these four measures are explained here in more detail.

### Inverse Efficiency Score

Townsend and Ashby (1978) were the first to introduce a combined speed-accuracy measure. They considered that the speed gain could be partly neutralised by correcting response time with the frequency of error occurrences. Considering performance of a subject *j* in a condition *i*, the *inverse efficiency score* (IES) is defined as

1 {\rm{IE}}{{\rm{S}}_{ij}} = \frac{{{\rm{R}}{{\rm{T}}_{ij}}}}{{{\rm{P}}{{\rm{C}}_{ij}}}}

where RT*_ij_* is the average *correct* RT of subject *j* in condition *i*, and PC*_ij_* is the proportion of correct responses (i.e., the converse of the proportion of errors committed by subject *j* in condition *i*: 1 – PE*_ij_*).

For example if correct RT is 900 ms and PC is 0.90, IES = 1000 (ms). In other words, IES is an RT measure corrected for the proportion of errors committed; the more errors, the larger the correction. That suggests that the score compensates RT for errors, and indeed under very specific conditions SAT can be neutralised in the IES score. Assume that RT can be changed via multiplication by a factor *m*, and that PC is changed in the same way, i.e., by multiplication of PC by the same factor. If the limiting condition holds that *m* > 0 and that m × PC <= 1 or *m* <= 1/PC, then

2 {{\rm{IES}}_{ij}} = \frac{{{{\rm{RT}}_{ij}} \times m}}{{{{\rm{PC}}_{ij}} \times m}} = \frac{{{{\rm{RT}}_{ij}}}}{{{{\rm{PC}}_{ij}}}}

which shows that under these conditions, IES is unaffected by this particular SAT modulation. However, because the choice response is usually limited to a number of possible alternatives, *m* × PC has no meaning when this value drops below the chance level *c* for a correct guess, so that it must be required that *m* × PC >= *c*. For example with binary choice responses *c =* 0.5, it follows that *m* × PC >= 0.5. Whether these assumptions including the limiting conditions can in reality be met, is open to doubt. For completeness, it seems useful to add that Townsend and Ashby (1983) warned not to use the measure when SAT is suspected to play a role.

### Rate Correct Score

Another measure, the *rate correct score* (RCS) was proposed almost thirty years later by Woltz and Was (2006). It is defined as

3 {{\rm{RCS}}_{ij}} = \frac{{{{\rm{PC}}_{ij}}}}{{{{\rm{RT}}_{ij}}}}

where RT*_ij_* is the average of *all* RTs of subject *j* in condition *i*. Note that if this measure would be based on only correct RTs it would be the inverse of IES (1/IES). Around the same time this measure was also proposed independently by Thorne (2006) under the name *throughput score*, and a variant using correct RT was already mentioned by Dennis and Evans (1996) under the name Ratio Index. Considering that by definition, RCS is the inverse of IES, it follows that RCS can be insensitive to SAT modulations under the same assumptions and limiting conditions as explained for IES.

### Linear Integrated Speed-Accuracy Score

Reasoning that the simple sum of RT and PE would yield a straightforward combined score if the measurement scales of both measures would be equal, Vandierendonck (2017) proposed the *Linear Integrated Speed-Accuracy Score* (LISAS). It is defined as:

4 {{\rm{LISAS}}_{ij}} = \left\{ {\begin{array}{*{20}{l}}
{{{\rm{RT}}_{ij}}}&{{\rm{if}}\ {{\rm{PE}}_{ij}} = 0}\\
{{{\rm{RT}}_{ij}} + {{\rm{PE}}_{ij}} \times \frac{{{S_{{{\rm{RT}}_{\rm{j}}}}}}}{{{S_{{{\rm{PE}}_{\rm{j}}}}}}}}&{{\rm{otherwise}}}
\end{array}} \right.

where {S_{{{\rm{RT}}_{\rm{j}}}}} and {S_{{{\rm{PE}}_{\rm{j}}}}} are the standard deviations of subject *j*’s RT and PE respectively. Multiplication of PE*_ij_* with the fraction of the standard deviations of RT and PE brings the two measurement scales to the same level. This score also takes only correct RTs to calculate RT*_ij_* and can be interpreted as RT corrected for the occurrence of errors.

Because this score is simply a weighted sum of the RT and PE measures, it follows that changes in RT can be compensated for by opposite changes in PE as long the weighted PE change is of the same magnitude as the RT change. A limiting condition is that the changed PE value remains positive and does not exceed the chance level for guessing.

### Balanced Integration Score

Still more recently, Liesefeld and Janczyk (2019) proposed the *Balanced Integrated Score* (BIS), which is defined as

5 {{\rm{BIS}}_{ij}} = {z_{{{\rm{PC}}_{ij}}}} - {z_{{{\rm{RT}}_{ij}}}}

where

6 {z_{{{\rm{PC}}_{ij}}}} = \frac{{{{\rm{PC}}_{ij}} - \overline {{\rm{PC}}} }}{{{S_{PC}}}}

and

7 {z_{{{\rm{RT}}_{ij}}}} = \frac{{{{\rm{RT}}_{ij}} - \overline {{\rm{RT}}} }}{{{S_{RT}}}}

Thus, this score is an additive combination of a standardized RT and a standardized PE measure. It is unfortunate that the authors have chosen to use the sample standard deviations of RT and PE because this results in a scoring relative to the sample mean, so that the same score in two samples actually has a different meaning. That this is not the best possible choice has been argued by Vandierendonck (2017, p. 660) in the discussion of the properties of the binning measure (Hughes, Linck, Bowles, Koeth & Bunting, 2014). Not only comparison of scores across samples but even the comparison of samples is made impossible because the average of sample-wide standardized scores is zero, so that all samples have a mean BIS score of zero. Apart from these comments, it is interesting to note that the BIS score is very similar to LISAS, for in a sample of a single subject, the BIS score is a linear function of LISAS; a proof for this is provided in Appendix A. For that reason, LISAS’s property of potential insensitivity to balanced changes in RT and PE does also apply to BIS with the same restrictions, be it that by using the sample standard deviations in the calculation also between-subject trade-offs seem to be possible. In other words, RT changes by one subject can be compensated by PE changes in another subject. It should also be noted that the authors claim that of the four integrated measures presented here, only BIS is relatively insensitive to SAT (Liesefeld & Janczyk, 2019, p. 56).

Another measure was proposed by Dennis and Evans (1996), namely the log A index (LAI), which uses the logit of the proportion correct responses (PC)

8 {\rm{logit}}({\rm{PC}}) = \log \frac{{{\rm{PC}}}}{{{\rm{PE}}}}

and is defined as

9 {\rm{LAI}} = \frac{{ - 1}}{{{\rm{RT}} - {{\rm{RT}}_{min}}}} \times \log \frac{{A - {\rm{logit}}({\rm{PC}})}}{A}

where RT*_min_* is the response time corresponding to chance responding (guessing), and A is the asymptote of the logit.^1^

### Theoretical Views of Speed-Accuracy Trade-Off

The pervasiveness and ubiquity of SAT in human (as well as in non-human) response data has incited the development of mathematical approaches to its study. Roughly speaking, two different approaches have been explored. In a first approach, some researchers have developed the idea that SAT involves a *mixing* of fast guesses with a high chance of being incorrect and slow controlled correct responses (e.g., Ollman, 1966; Yellott, 1971). Although such models have the advantage to account for the (rare?) presence of bimodal response distributions (see e.g., Dutilh, Wagenmakers, Visser & van der Maas, 2011), they predict a linear relationship between speed and accuracy, and such a relationship is at best correct for a subrange of the RTs and PEs considered (Heitz, 2014; Stafford et al., 2020). Interestingly though, based on these models and their distinction between fast guesses and stimulus controlled responses, Yellott (1971) has developed a method to estimate the average latency of stimulus controlled responses (SCR), namely

10 {\rm{SCR}} = \frac{{{\rm{PC}} \times {{\rm{RT}}_c} - {\rm{PE}} \times {{\rm{RT}}_e}}}{{{\rm{PC}} - {\rm{PE}}}}

where RT*_c_* and RT*_e_* refer to average correct and incorrect RT, respectively. A series of experiments varying response deadlines showed that SCR, although RTs and PCs varied over the conditions, was remarkably invariant over the different conditions and experiments. Note that the formula in Eq (10) yields another measure that integrates speed and accuracy, that is included in the present simulations.

The alternative view on SAT assumes that a choice response requires the accumulation of (sensory) information in support of the response alternatives. A response is then initiated as soon as a sufficient amount of information has been accumulated. Several mathematical models have been proposed to embody such a *sequential-sampling* process that typically involves a random walk (Fitts, 1966), or a drift diffusion process (Ratcliff, 1978), but other modeling variations have been published (see Heitz, 2014, for a review).

An elaboration of all these models is beyond the scope of the present endeavour. Because Ratcliff’s Drift-Diffusion model (DDM) is the most widely used of these sequential-sampling models and several data-analytic tools are available to estimate the model’s parameters from the data (Vandekerckhove & Tuerlinckx, 2007, 2008, Voss & Voss, 2007; Wagenmakers, van der Maas & Grasman, 2007), and furthermore also methods to simulate the model have been developed and tested (Ratcliff & Tuerlinckx, 2002; Tuerlinckx, Maris, Ratcliff & De Boeck, 2001), DDM will be used for the present simulations.

According to Ratcliff (1978)’s diffusion model, sensory input provides information that is accumulated over time. The amount of information randomly drifts between two boundaries, a lower boundary representing the incorrect response choice and an upper boundary for the correct response choice. When after some time the amount of accumulated information has been wandering between the two boundaries and then crosses one of these boundaries, the corresponding response is initiated. The average drift rate (towards the upper boundary), the variability (noise) of the drift rate, and the distance to the boundaries are the variables (parameters) that determine the time needed to reach one of the boundaries, the response time. If the distance to the boundaries becomes smaller (keeping drift rate and noise fixed), response time will become shorter, but the chances for an error will become larger. Conversely, if the distance to the boundaries becomes larger (again with the same drift rate and noise), response time will increase, but the likelihood of an error will decrease. So, basically in this model (as well as in the other models of this class of sequential-sampling models, see Heitz, 2014; Stafford et al., 2020, for more details), the position of the boundary determines SAT: raising the inter-boundary distance increases response time and decreases proportion of errors, whereas lowering the inter-boundary distance has the converse effect.

Apart from these developments that are mainly situated in the experimental research tradition, SAT has also been a concern in psychometric research. Without attempting to give a representative overview, a few examples of such research can be found in Van der Linden (2007), H.L.J. van der Maas, Molenaar, Maris, Kievit and Borsboom (2011), H.L.J. van der Maas and Wagenmakers (2005). This approach focuses on scoring rules involving both speed and accuracy (e.g., Maris & Van der Maas, 2012).

Since the introduction of the first SAT models in the 1960’s an enormous amount of scientific progress has been made. Considering the availability of ready-to-use tools for parameter estimation (e.g., Voss & Voss, 2007; Wagenmakers et al., 2007), some authors have argued that the field has made so much progress that the focus should be shifted from using RT and PE to using the model parameters to account for these observables (Heitz, 2014; Stafford et al., 2020). True as this may be for people working on SAT modeling, for researchers with other research questions in mind, this may be a difficult choice, because it is at present not clear which of the models they should prefer to interpret the data, and whether the findings established with one model are generalizable to another model. Furthermore, for appropriate parameter estimation more data are needed than typically collected in hypothesis testing experiments. It is, however also possible to take a still broader perspective, such as the one promoted by researchers in the field of cognitive control processes. The choice for a particular SAT criterion by a person is one of the many control actions that can be made in addition to orientation of attention, breadth of attention, choice of response modality, etc. (e.g., Botvinick, Braver, Barch, Carter & Cohen, 2001; Logan & Gordon, 2001). Within this perspective, SAT settings are flexible and can be changed on the fly in a reactive way to the presence of situational characteristics.

So, it is not only possible to instruct or to encourage participants in an experimental setting to choose a particular SAT approach (e.g., favouring speed over accuracy), participants evaluate whether their SAT approach is adapted to the current situation and accordingly change their approach for the upcoming events (cf. the findings with respect to the flanker task Gratton, Coles & Donchin, 1992).

In other words, even if SAT settings are selected prior to execution of a series of similar tasks (e.g., by instruction), during execution of the tasks, SAT will be continuously adapted. Hence, when easy and difficult versions of tasks are presented blockwise, it is quite likely that the participant will select appropriate SAT settings for each block of tasks, increasing the chances of a confound between SAT and task difficulty effects. For example, by speeding up responses to easy tasks, more errors will be committed to this type of tasks, and by securing accuracy in the difficult tasks, these will be executed slower but more accurately, with paradoxical outcomes as a result. This can easily be avoided by mixing easy and difficult trials: when at the occasion of easy trials, the participants decide to go faster, it is equally likely that the effect of the changed SAT setting will affect an easy or a difficult trial. Clearly, such a mixed design spreads the effects of the SAT adaptations over the different task difficulty conditions, *de facto* neutralising these adaptations.

The usage of mixed conditions within-subjects designs is well in line with habits in cognitive psychological research. In the broader context where all kinds of control actions can occur, experiments typically use such repeated measures designs because the experimental conditions are continuously varied in a random or pseudo-random sequence of trials. Indeed, conflict tasks such as the Stroop task, the flanker task, and the Simon task, cannot be presented block-wise: a comparison of performance on a block of congruent Stroop trials and a block of incongruent Stroop trials would be uninformative because such conditions invite the participants to apply reactive control strategies to ameliorate performance (Gratton et al., 1992). Apart from such conflict tasks, many other tasks used by cognitive psychologists have similar properties. These tasks include inhibition tasks such as go/no-go, anti-saccade, stop-signal, variations on task switching procedures such as alternating runs, cued switching, many kinds of judgment tasks such as lexical, semantic, alphabet and numeric decision tasks, and many others.

With such mixed condition repeated measures design, condition-wise SAT choices are less likely to occur, although reactive SAT modulations cannot be excluded. That does not mean that the problems related to SAT do not exist, but it indicates that the problem is transformed from the question of deconfounding experimental and SAT factors to the question whether experimental factors that are not directly under the influence of variable SAT settings can be properly detected and whether the observed speed and accuracy can be combined to come to valid conclusions.

Phrased differently, if an experimental factor of interest is present and randomized over a sequence of trials, can the observed effects related to this experimental variable be trusted if the task is performed under different SAT settings? This particular question is investigated in the present article by using a repeated measures design with an experimental variable (e.g., control vs. experimental condition) crossed with an SAT variable representing different settings (e.g., speed stress, neutral stress, accuracy stress). In what follows, this experimental variable or variable of interest will be referred to as Test. A Test by SAT Settings design was used in the simulation studies reported here. In such a design, the Test conditions are randomized over a series of trials or events and in each of these trials (or events) RT and PE are registered and used to calculate combined measures. Moreover, the basic design was implemented with variations in the overall average PE and the size of the SAT modulation. This approach was based on the considerations (a) that combined speed-accuracy measures are increasingly used to assess cognitive task performance, (b) that some of these measures entail a limited promise of being insensitive to SAT, and (c) that claims have been formulated about the usefulness of some measures when SAT is involved. The present series of simulation studies was designed to investigate the validity of these promises and claims.

None of the studies will involve a between-subject manipulation or between-subject SAT modulation because such a design may be confounded by condition-wise SAT modulations, and perhaps even more important, because such a design allows for between-subject trade-offs: the increased speed by one subject may be compensated by the increased accuracy of another subject. In the end, this does not help to better understand how SAT adaptations based on decisions made by the participant affect other aspects of performance.

Four studies are reported. The first study addresses the expectation that LISAS and BIS which are both linear measures are insensitive to SAT if the SAT effects are completely linearly balanced. The second study uses SAT variations in line with (Ratcliff, 1978)’s DDM to test whether any of the combined measures are insensitive to SAT. Apart from proactively controlled SAT, it has already been suggested that in the kind of tasks which are the focus of the present project, reactive SAT modulations do occur. The third study investigates whether the integrated speed-accuracy measures are insensitive to such reactive modulations as defined within the context of DDM. Finally, because it has been suggested that in particular circumstances, discontinuous or two-phase approaches to SAT are used (e.g. Dutilh et al., 2011), the fourth study investigates the sensitivity to such discontinuous SATs. In all these studies^2^ the four integrated speed-accuracy measures described in the introduction, namely inverse efficiency score, rate correct score, linear integrated speed-accuracy score and balanced integrated score, as well as the stimulus controled response latency proposed by Yellott (1971) will be applied to simulated response data. The questions of interest are:

1. Are any of these measures insensitive to SAT settings and if so under which conditions?
2. Do these measures efficiently detect the experimental effect (factor Test) irrespective of the presence and the amount of SAT modulations?

## Study 1: Balanced Speed and Accuracy Effects

As explained in the introduction, among the combined speed-accuracy measures, two are based on the principle of assigning equal weight to the speed and accuracy components of the measure, namely LISAS and BIS. These two measures assume a linear relationship between the composite speed and accuracy measures and should therefore be insensitive to linear SAT; more specifically, this implies that the size of the speed modulation is compensated by an accuracy modulation of the same size in the opposite direction. Given that the class of models which underly the development of the SCR measure equally assume linear speed-accuracy compensations, it might be expected that this score would also be insensitive to the linear SAT variations. Linearity was implemented by using an atheoretical approach in which the effects on speed (response time, RT) and accuracy (proportion of errors, PE) were defined in a structural statistical (anova) model. At the same time, the effects of an independent experimental variable should still be efficiently detected by these integrated measures (and in fact also by integrated measures that are sensitive to SAT). Because the experimental variable is orthogonally crossed with the factor that involves SAT differences, the integrated measures are not expected to detect an interaction of the experimental factor with SAT. Study 1 was designed to test all these expectations.

### Method

In order to achieve balanced effect conditions, a two-factor design was defined with one difficulty factor and one speed-accuracy factor. The difficulty factor (Test) had two conditions, namely an easy or control condition (relatively fast with low error proportion) and a difficult or experimental condition (relatively slow with high error proportion), so that there is a positive correlation between the RT and the PE means. The speed-accuracy factor had three levels: speed stress (fast and error-prone), neutral (moderate speed and errors), and accuracy stress (slow and lower error rate). In other words, this factor has a negative correlation between the speed and the accuracy means. In what follows, this factor is referred to as SAT Settings to stress that it varies the settings of the trade-off. This design corresponds to the structural model described in equation 11.

11 {Y_{ijk}} = \mu + {\alpha _i} + {\beta _j} + {\pi _k} + {\in_{ijk}}

where *Y* is the dependent variable, *μ* its mean, *α_i_* the task difficulty effect in condition *i, β_j_* the speed-accuracy adaptation in condition *j, π_k_* variability associated with subject *k* and *Є* an ex-gaussian distributed (in the case of the speed variable) random error; for accuracy, the distribution is binomial. This structural model and the assigned parameter values for speed and accuracacy as shown in ***Table 1*** is used here in the first place as a guidance to data generation in Study 1. Note that this model has no term for the interaction of the Test and SAT factors, and that the distributions of PE are proportional to RT, except for the ex-gaussian component on the RT distribution.

**Table 1:** Values assigned to the structural model parameters for response time and proportion errors in Study 1. Note that the distribution parameters are proportional across the two dependent variables.


PARAMETER	RT	PE

*μ*	1000	0.20

*σ*	160	0.40

*σ_p_*	8	0.02

*α_i_*	± 5	± 0.0125

*β_j_*	[–20, 0, 20]	[0.050, 0, –0.050]

*π_k_*	N(0, *σ_p_^2^*)	N(0, 0.1414)

*Є_ijk_*	exN(0, *σ^2^*, 50)	coded 1/0 (binomial)


*Note*: The symbols *σ* and *σ_p_* refer to respectively the population standard deviation and standard deviation of the subject-associated effect (which is *σ* * 0.05). No value is provided for Ɛ in the column PE because accuracy is coded 1/0 and the value is determined by a uniformly distributed random value based on the cell mean; aggregation of the accuracy scores per condition results in a variable with a binomial distribution. N(x, y) denotes a gaussian distribution with mean x and variance y; exN(x, y, z) denotes an ex-gaussian distribution with a gaussian part N(x, y) and an exponential distribution with mean z.

In the simulation study, this design was replicated as a function of variations in two factors: (a) the SAT effect (*β_j_*) on dependent variable RT was varied from 2 to 20 in steps of 2, and on dependent variable PE it was also stepwise varied from 0.005 to 0.05 in steps of 0.005, (b) the overall (average) PE (defined as 0.20 in ***Table 1***) was varied in 4 steps (0.05, 0.10, 0.15 and 0.20) so as to cover a broad range of proportion of errors. At each of these four PE sizes, the ratio of the effect size and the PE standard deviation was the ratio of the RT effect size and the RT standard deviation in order to keep the RT and PE changes in balance. For example, if overall PE was 0.10, the standard deviation was 0.30, and *β_j_* on PE was 0.3/160 × 20 = 0.0375 instead of 0.05 as shown in ***Table 1***. Each application was based on 100 statistical subjects, with 200 trials per cell. The dependent variables RT and PE were registered for each trial in each condition of each statistical subject. From these data also the integrated speed-accuracy measures defined in the introduction were calculated, namely IES, RCS, LISAS, BIS and SCR. For these simulations, as for the other studies reported in the present article, random generation functions provided by the Gnu statistical library (gsl) were used (Galassi et al., 2019).

The collected data were subjected to repeated measures analyses of variance on the 2 (Test: Control vs. Experimental) × 3 (SAT Settings: Speed stress, Neutral or Accuracy stress) design by means of multivariate general linear modeling with contrasts in the dependent variables. For each of the measures, a separate analysis was performed. F-values were estimated from Wilk’s *λ*. In addition, the effect sizes were estimated by calculating partial eta-square (\eta _p^2). These analyses were based on the structural model specified in Eq (11). In order to verify the expectation of no interaction of Test and SAT settings, the interaction term was included.

### Results and Discussion

In order to give the reader some feeling for the data collected in this simulation with 40 replications of the 2 *×* 3 design, Appendix B more extensively reports the results in one of the samples, namely the sample based on PE = 0.20 and the maximal SAT effect (20/0.05). The appendix reports the analysis with a conventional degree of detail.

**Figure 1:**
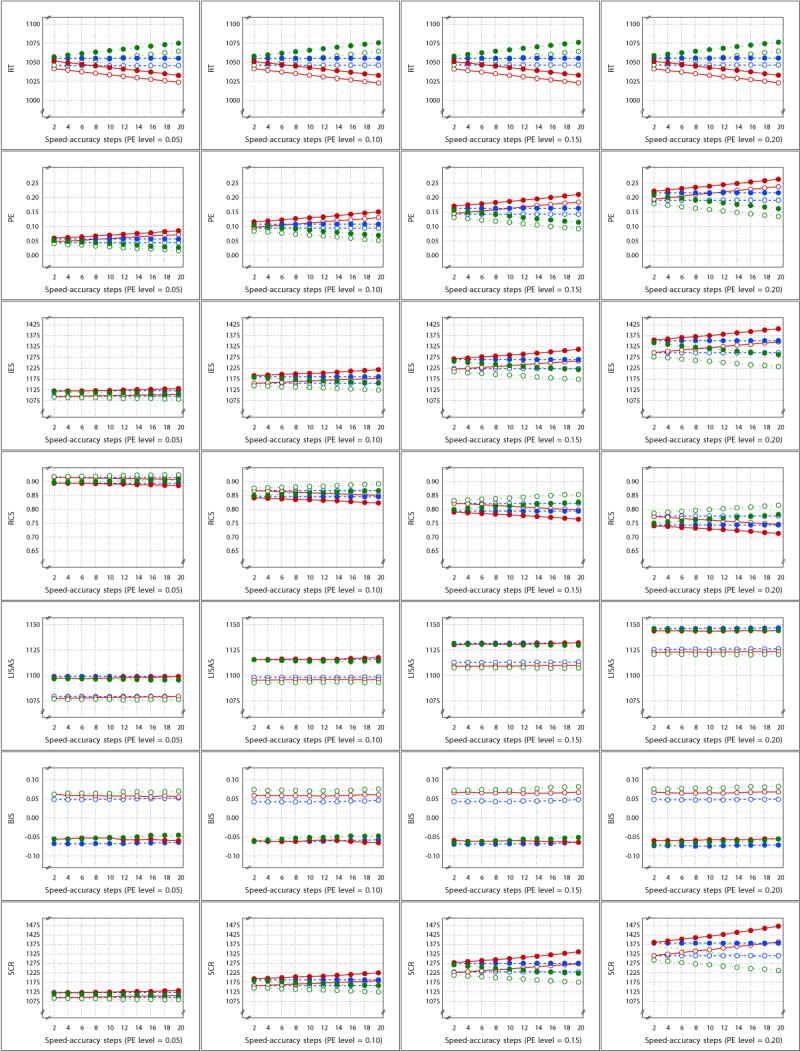
Sample Means in Study 1 as a function of Test × SAT Settings × SAT size (x-axis in each panel) × PE level (panels from left to right). Each row of panels shows the result for a single dependent variable. The curves within each panel show the cell means of the Test × SAT Settings combinations. Legend: Open circles for control condition and closed circles for experimental condition; red solid lines for speed stress, blue dashes for neutral SAT, and green dotted lines for accuracy stress.

These 40 (4 × 10) variations of the basic design allow to investigate whether the SAT effect is detected by all measures irrespective of the size of the SAT effect and also irrespective of the degree to which response errors tend to occur. ***Figure 1*** shows the means of the cells of the basic 2 by 3 design for each of the measures in the study as a function of the four PE levels and the ten SAT sizes. This figure confirms that PE varied over the four PE levels (second row of panels), but RT remained constant (top row); they both varied as a function of the conditions in the design and the SAT sizes, suggesting that with larger SAT sizes, the difference between speed and accuracy stress became stronger. These panels also show that the curves representing the control and experimental condition were completely parallel within the SAT Settings conditions. The IES and RCS measures (rows 3 and 4) confirmed this pattern, while LISAS and BIS (rows 5 and 6) showed no change over the SAT sizes, but the Test condition difference remained stable over all variations in SAT and PE. Although the SCR means (row 7) also show parallel curves within the SAT conditions and larger SAT differences with increasing SAT size, it should be noted that in comparison to RT, the order of the effects is reversed, with larger SCR for speed stress and lower SCR for accuracy stress.

**Figure 2:**
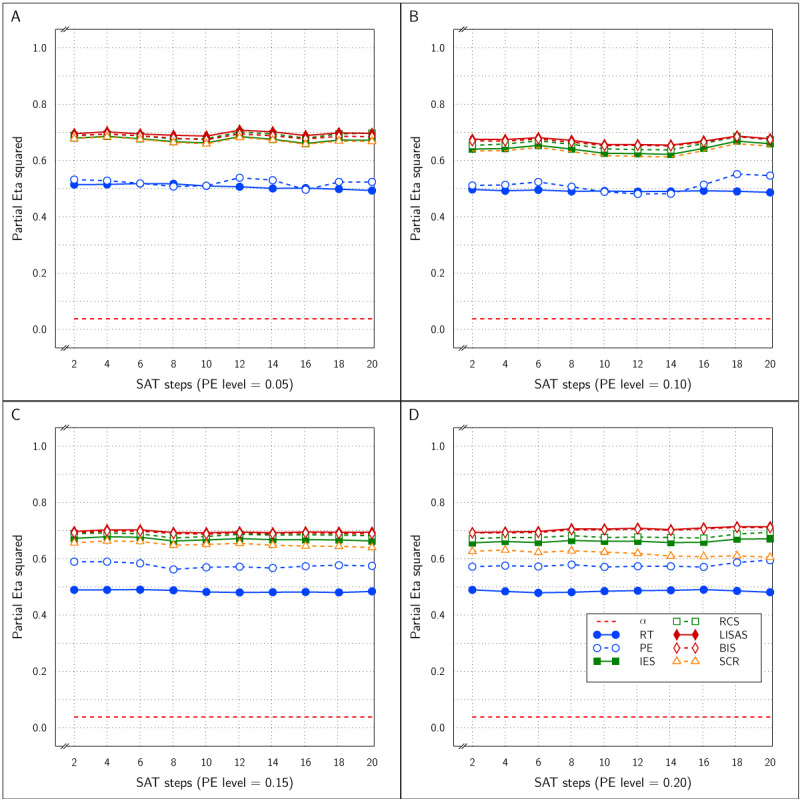
Estimated effect size (\eta _p^2) of the Test effect as a function of the variation in PE (panels A to D) and in SAT size for RT and PE and for the five combined measures in Study 1.

***Figure 2*** displays the observed Test effect size (\eta _p^2) at the levels of PE for all six measures. Note that the Test effect was of medium size (d = 0.6) and constant. It shows that the Test effect was reliably detected by all the measures irrespective of the overall amount of error committed. The figure also shows that the PE level did not affect the size of the Test effect; this confirms expectation because the RT effect size was constant over all instantiations of the design, while the PE effect size was proportional to the PE variance and thus kept constant. The size of the SAT effect did not affect the detected Test effect size in any of the measures and the five integrated measures were about equally efficient at detecting the Test effect and all five detected the effect more efficiently (larger effect size) than did RT and PE on their own, which is consistent with findings reported by Vandierendonck (2017).

**Figure 3:**
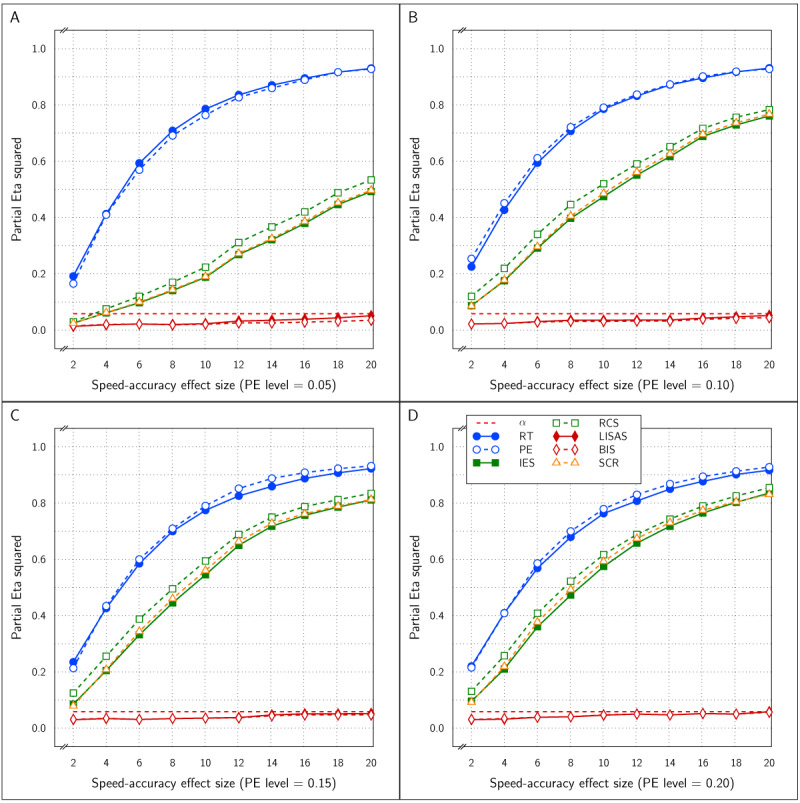
Estimated effect size (\eta _p^2) of SAT Settings as a function of PE level and SAT strength for RT and PE and the five combined measures in Study 1. The dashed line (labeled *α*) represents the significance threshold for these data.

The effect sizes of the factor SAT Settings as a function of the manipulated SAT effect size and overall PE is shown in ***Figure 3***. The four panels which correspond to the four PE levels show that as the SAT effect size increased, the observed effect size in RT and PE grew towards an asymptote above an \eta _p^2 value of 0.90 and did not differ as a function of the size of overall PE. As expected, the SAT effect detected by the combined measures LISAS and BIS did not vary over the manipulated SAT effect size and stayed at a level below the 0.05 significance threshold. For the other three combined measures (IES, RCS, and SCR) the observed effect size grew with the SAT size but stayed much lower than the constituting measures RT and PE; they attained an asymptote for \eta _p^2 around 0.5 to 0.8 depending on the overall PE size: the higher average PE, the higher the asymptote.

Except for SCR, these findings are in agreement with the expectations, but before discussing this in detail, it must be shown that the verification conditions for this Study were met.

1. First, The RT and PE measures reliably detected the effect of the Test factor irrespective of the size of PE and irrespective of the size of the modeled SAT effect.
2. These two basic measures also reliably detected the effect of SAT Settings. ***Figure 3*** shows that in each panel even at the lowest SAT size, the effect was above the significance level (red dashed line).

With respect to the expectations about the combined measures, it is clear from ***Figure 2*** that they all efficiently detected the Test effect and were more efficient than RT and PE separately. With respect to the SAT Settings effect, IES, RCS, and SCR efficiently detected this effect but the efficiency depended on the overall PE size and the size of the SAT effect itself. Moreover, it can be said that these measures are substantially less efficient than RT and PE themselves to recover the SAT effect, suggesting that these measures to some degree compensate opposite RT and PE changes. Under the conditions of the present study, the other two combined measures, LISAS and BIS, were completely insensitive to the SAT effect irrespective of its size, as expected. In all four panels of ***Figure 3***, the observed effect size was near zero and stayed below 0.059 which corresponds to the point at which the effect size attains significance. Finally, the interaction of the Test and SAT Settings factors was not reported thus far, but as can be seen in ***Figure 1*** the lines representing the two conditions are basically parallel, and in fact in neither of the 40 simulations, a trace of interaction could be seen (all *p* were far above the significance threshold). The implication of this lack of interactions in combination with the strong main Test and SAT Settings effects is that for all measures the Test effect was present and reliable at each SAT Settings level and that the SAT Settings effect was present and reliable within the control and the experimental condition.

So, it can be concluded that except for SCR, the expectations were all confirmed. This first study hence suggests that it is for possible for LISAS and BIS to be insensitive to a SAT manipulation which is balanced across speed and accuracy in a repeated measures design. That the SCR latency was not insensitive to the SAT trade-offs and that the order of the effects was reversed suggests that the logic behind this measure does not seem to be valid for these linearly balanced trade-offs.

## Study 2: SAT Effects based on the Drift-Diffusion Model

The aim of the second study was to use the same basic 2 × 3 Test by SAT Settings design with parameter settings derived from Ratcliff (1978)’s DDM. In all other respects, the planned study was similar to Study 1.

As explained by Tuerlinckx and colleagues (Ratcliff & Tuerlinckx, 2002; Tuerlinckx et al., 2001) simulation of the DDM can be quite tedious. They describe and discuss the utility of several approaches to simulate the model. Their results show that usage of a discrete random walk model with very small time steps to approach continuity is not only an acceptable, but is also useful though mathematically simple approach, and when the time steps are sufficiently small, the method yields a very close approach to the drift-diffusion model. This approach was used in the present simulations.

A random walk is a sequence of steps of a fixed size that after some time crosses either an upper (correct response) or a lower boundary (incorrect response). Each step has a time duration *h* and a size *δ* in the direction of one of the boundaries. A step in the direction of a correct response is taken with probability *p* and a step in the other direction has probability *q =* 1 *– p*. In the diffusion process, the accumulation of evidence in favour of the correct response, proceeds with drift rate *ν*; *s* is the standard deviation of the drift rate (the degree of noisiness); the upper boundary is *a* and the lower boundary is 0. The process starts at *z* (0 *< z < a*) (typically *z = a/*2). In the limit when *δ* → 0, *h* → 0, the random walk approaches the diffusion model, as specified by the following equations (Ratcliff & Tuerlinckx, 2002):

12 \nu = (p - q)\delta /h,

13 {s^2} = 4pq\frac{{{\delta ^2}}}{h},

14 \delta = s\sqrt h,

15 q = 0.5(1 - \nu \sqrt {\frac{h}{s}}),

The diffusion parameters are related to speed and accuracy in the following way:

1. As *ν* (drift rate) increases, both RT and PE decrease, so that *ν* can be considered to indicate task difficulty (the smaller *ν* becomes, the more difficult the task is in terms of slower and more error-prone performance);
2. As *a* (boundary separation) increases, RT also increases, but PE decreases, resulting in a trade-off between speed and accuracy and therefore *a* is commonly considered to be parameter driving speed-accuracy trade-off;
3. As *s* (drift rate noise) increases, RT decreases but PE increases, which also contributes to trade-off.

Interestingly these parameters complemented with non-decision time (*Ter*) are the only parameters of interest in the “simple” version of DDM as developed by Wagenmakers et al. (2007). In their EZ Diffusion model, *s =* 0.10 and is fixed, *ν, a* and *Ter* are computationally derived from three statistics, namely mean RT, variance of correct RT and proportion of correct responses. These properties provide a means for checking the validity of the random walk approach: by calculating these three statistics on the simulation results from the random walk approach and feeding them into the EZ Diffusion model formula’s, the original parameters used in the random walk should be recovered or at should least be sufficiently close to the original values. In order to be able to take advantage of these properties, *s* was fixed at 0.10 and *z = a/*2 in the simulations reported here.

## Method

Following Ratcliff and Tuerlinckx (2002), the simulations were performed with *h =* 0.05 ms (see also Tuerlinckx et al., 2001). For the present set of simulations, a 2 (Test: control vs. experimental) × 3 (SAT Settings: speed stress, neutral, accuracy stress) design was implemented. The same overall means as in Study 1 were targeted, namely mean RT ⋍ 1000 ms (i.e., 700 ms response time + a fixed non-decision time of 300 ms)^3^ and mean PE was varied in four levels as in Study 1, namely 0.05, 0.10, 0.15 and 0.20. A parameter search was performed to select appropriate values for the parameters. First appropriate values for *ν* and *a* were selected to achieve the four error levels with RTs close to 1000 (including the non-decision time). The selected values for *ν* were 0.135, 0.105, 0.090 and 0.070 for the error rates of respectively 0.05, 0.10, 0.15 and 0.20; the corresponding *a* values were 0.22, 0.20, 0.19 and 0.18. Next, the Test effect (easy-difficult) was obtained by taking *ν* + 0.002 in the control condition and *ν* – 0.002 in the experimental condition. For the SAT settings factor, the values *a* – 0.001*n, a*, and *a* + 0.001*n* were used for respectively speed stress, neutral, and accuracy stress, where *n* represented the number of steps specified in the size of the SAT manipulation across the simulations. Basically, the structural model of Eq (11) as defined for Study 1 is also applied in the present Study with parameter values for *α* and *β* defined indirectly by setting appropriate diffusion parameters. Again, no definition is included for the interaction of the factors Test and SAT settings. This does not mean that the resulting speed and accuracy variables will not interact; only that there is no theoretical basis for an a priori definition of the interaction. As in Study 1, the interaction is tested in the data analysis.

A repeated measures design was used with 40 statistical subjects.^4^ Similar to Study 1, the present study involved 4 (PE levels: 0.05, 0.10, 0.15, and 0.20) × 10 (SAT levels: 1–10) variations of this 2 × 3 design.

### Results and Discussion

Before reporting all the findings in detail, first checks on the validity of the simulations are addressed. The results of each simulation were used to test whether the EZ Diffusion model returned similar parameter values. For each statistical subject in each of the simulations, mean RT, variance of correct RTs and proportion correct responses were calculated and used to obtain the EZ Diffusion parameters. Let *s_i_* be the parameters used in the simulation and let *e_i_* be the parameters obtained from the EZ Diffusion method, a χ*^2^* value was obtained

16 {\chi ^2} = \sum\limits_i {\frac{{{{({s_i} - {e_i})}^2}}}{{{s_i}}}}

where *i* refers to respectively the parameters *ν, a*, and *Ter*. The χ*^2^* values obtained in each of the 6 conditions (2 × 3 design) of the 40 statistical subjects in all 40 (4 × 10) simulations were smaller than 1 and had a probability under H_0_ of at least 0.99 confirming that the deviations between the used and the returned parameter values were very small to nonexistent.

**Figure 4:**
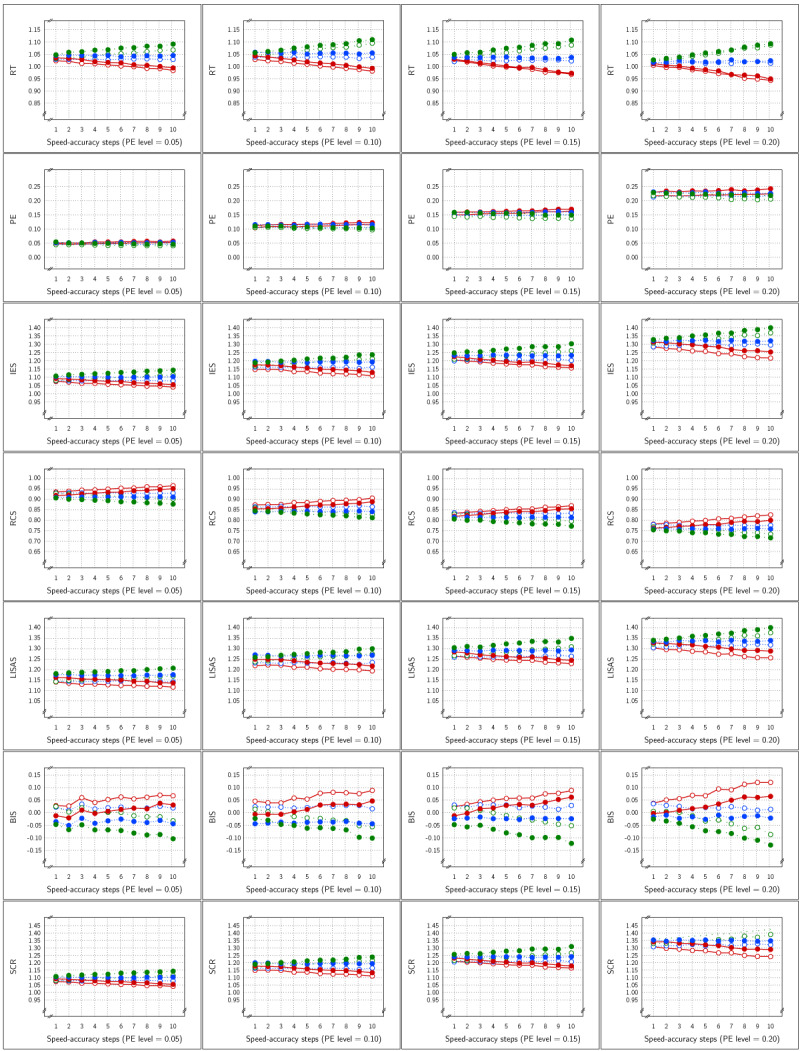
Sample Means in Study 2 as a function of Test × SAT Settings × SAT size (x-axis in each panel) × PE level (panels from left to right). Each row of panels shows the result for a single dependent variable. The curves within each panel show the cell means of the Test × SAT Settings combinations. Legend: Open circles for control condition and closed circles for experimental condition; red solid lines for speed stress, blue dashes for neutral SAT, and green dotted lines for accuracy stress.

The means per cell of the basic design are presented in ***Figure 4*** as a function of PE levels and SAT size. For all measures, this figure shows SAT effects that increased with the size steps. For all measures, the Test effect was rather stable over SAT size steps and PE levels, but the effect seemed to be rather small in PE. In contrast to Study 1, the SAT effects in SCR were not reversed.

**Figure 5:**
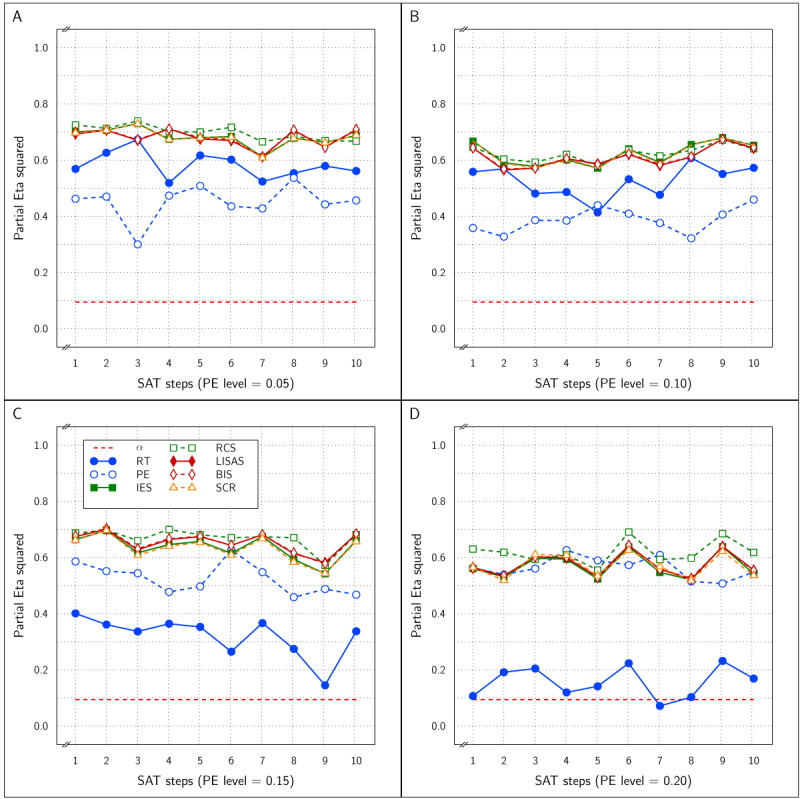
Estimated effect size (\eta _p^2) of the Test effect as a function of the variation in PE (panels A to D) and in SAT size for RT and PE and for the five combined measures in Study 2.

***Figure 5*** presents the size of the estimated Test effect (\eta _p^2) as a function of overall PE and SAT size. Similar to Study 1, the effect size for RT decreased with average PE level, while at the same time the PE effect size tended to increase. In almost all samples, the estimated effect size for all five combined measures was larger than that for RT and PE.

**Figure 6:**
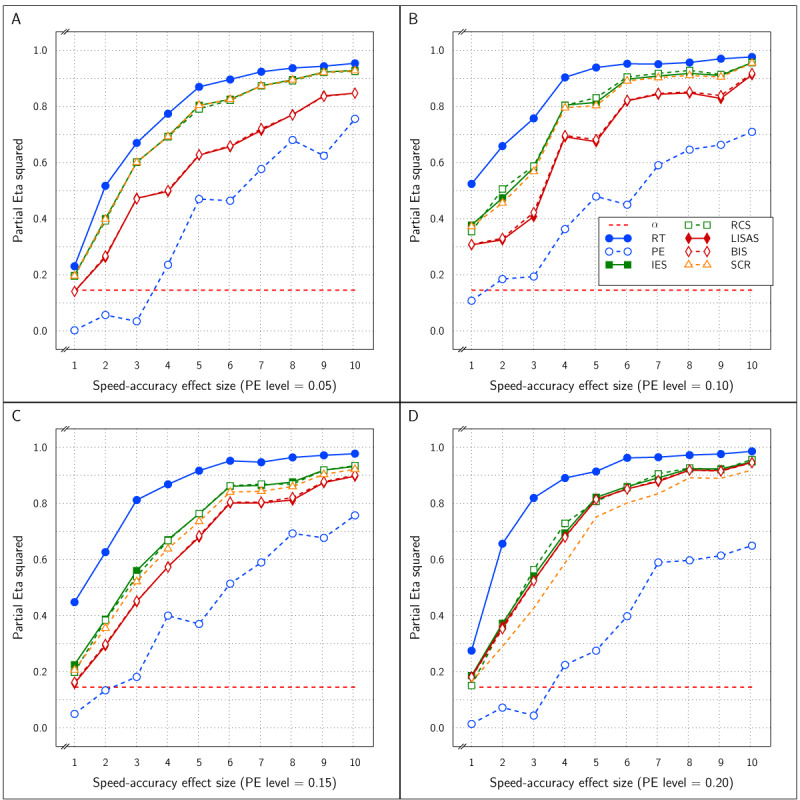
Estimated effect size (\eta _p^2) of SAT Settings as a function of PE level and SAT strength for RT and PE and the five combined measures in Study 2. The dashed line (labeled *α*) represents the significance threshold for these data.

***Figure 6*** displays the estimated effect of the SAT Settings factor as a function of the size steps for all six measures. The four panels of the figure show the relationship with respect to the four levels of PE. Overall, RT was most sensitive and PE was least sensitive to the SAT effect. At the lower PE levels, IES, RCS and SCR were almost as sensitive to the SAT effect as RT, and more sensitive than LISAS and BIS; at the higher PE levels, sensitivity of the five integrated measures to SAT was less than that of RT but the differences between these measures were very small. As in Study 1, it seems that compensations of opposite changes in RT and PE are slightly but not completely compensated in the integrated measures. There was some variation in sensitivity across the four PE levels, although the results seemed quite stable except for LISAS and BIS which increased with PE level. It thus appears that irrespective of the average PE level, all integrated measures yielded strong and significant effect size estimates. For the smallest effect size, this corresponds to on average of 7 ms faster or slower than the neutral condition, with an average PE increase or decrease of 0.0014 in comparison to the neutral condition. This is clearly a situation in which the size of the RT effects are not in balance with the PE effect sizes in a trade-off. In other words, to the extent that the diffusion model correctly describes SAT, none of the measures considered is insensitive to this trade-off.

By design Study 1 did not include any interactions for the factors Test and SAT Settings and indeed no interactions were observed in that study. Whether these factors interact in the present study depends on the degree of imbalance between RT and PE modulations and the size of the modulations. In fact, the range of SAT sizes was very large and with size the asymmetry in RT increases. As a consequence, the factors Test and SAT Settings interacted in a subset of the samples: in the 40 samples, the interaction was significant once (RCS), 4 times (PE), 5 times (RT), or 6 times (all other measures). Importantly, the question is whether these interactions limit the conclusions that can be drawn about the effect size of the factor Test in this design. In order to test this, planned contrasts were used to test the size of the Test effect separately in each of the three SAT Settings conditions. These findings are not presented here at large, but it was observed that in all samples the Test effect was reliably detected in each of three SAT Settings for all five integrated measures (between 98.3% and 100% of the 120 tests were significant); the \eta _p^2 values observed were between 0.08 and 0.61 for IES between 0.13 and 0.61 for RCS, between 0.09 and 0.62 for LISAS and BIS, and between 0.08 and 0.61 for SCR.

The results of the present study are at variance with those of Study 1; whereas Study 1 showed that LISAS and BIS are insensitive to SAT when there is a linear relationship between speed and accuracy, the present study shows that this finding cannot be generalised to a context where the relationship between speed and accuracy is clearly nonlinear. This finding also seems to contradict the claim formulated by Liesefeld and Janczyk (2019) about BIS’s insensitivity to SAT. This will be addressed in the General Discussion section. Overall sensitivity to SAT modulations, notwithstanding, the Test effect remained present in all SAT variations and was reliably detected by all the integrated measures.

## Study 3: Reactive Speed-Accuracy Modulations

Thus far effects associated with proactive adaptations to speed and accuracy were considered. According to the conflict-monitoring hypothesis of Botvinick et al. (2001), reactive as well as proactive cognitive control is driven by the degree of conflict present in the situation. One manifestation of such reactive control is the observation (e.g., Laming, 1968) that immediately after an error, RT increases and on the trials that follow, RTs slowly decrease again moving towards average. Likewise, immediately after an error, PE decreases and on the following trials PE tends back to average.

The present study examined whether such reactive speed-accuracy adaptations which are clearly present in RT and PE are also detected by the integrated speed-accuracy measures. In the mixed conditions repeated measures context used in the present studies, an adaptation after an error may as well affect performance in a consecutive control as a consecutive experimental trial. Hence, these reactive SATs are not condition-specific and it is not possible to distinguish a priori between modulations increasing speed and those increasing accuracy. Therefore the basic design was adapted to have an SAT factor with only two levels, resulting in a 2 (Test: easy vs. difficult trial) × 2 (SAT: no adaptation present vs. reactive adaptation) with the underlying rationale that the label “easy” refers to trials that have low conflict, are compatible, congruent or involve set-repetition and the label “difficult” refers to their opposites: high conflict, incompatible, incongruent or involving a set switch.

### Method

Except for the usage of a 2 by 2 repeated measures design, the same method was applied as in Study~2 based on the diffusion model. A 2 by 2 repeated measures design was implemented on samples of 40 statistical subjects that varied in average PE (4 levels: 0.05, 0.10, 0.15 or 0.20) and the strength of the SAT modulation (10 steps). The same diffusion model parameters as in Study 2 were used.

### Results

**Figure 7:**
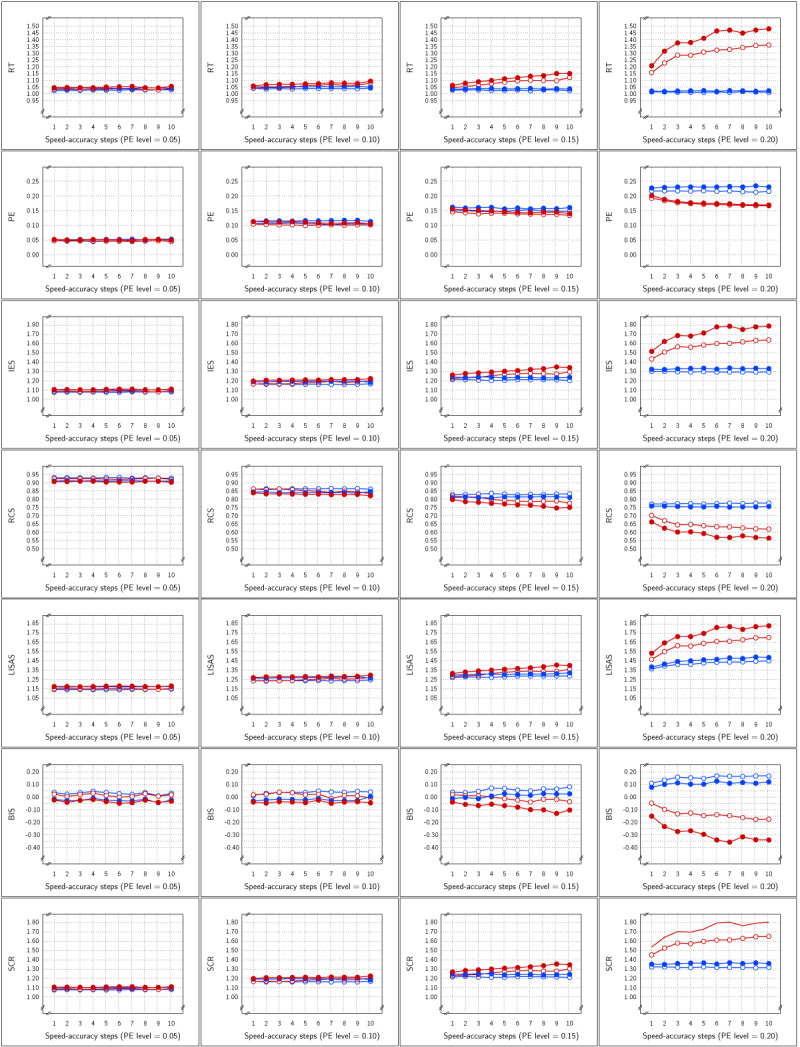
Sample Means in Study 3 as a function of Test × SAT Presence × SAT size (x-axis in each panel) × PE level (panels from left to right). The cell means of the Test × SAT part of the design are shown in the curves within each panel. Each row of panels shows the result for a single dependent variable. Legend: blue for SAT-absent conditions, red for SAT-present conditions; open circles for control condition, closed circles for experimental condition.

The means per cell are displayed in ***Figure 7*** as a function of PE average and SAT strength for each of the seven measures in the study. For all measures, the figure illustrates that the SAT modulations were quite small when the PE level was rather low, which follows from the fact that with smaller error proportions, the frequency of error-driven speed-accuracy modulations will be lower, resulting in a smaller average amount of adaptation. As PE level increased, and thus also the chance of an error, the larger the SAT modulation observed. Similar to Study 2, SAT effects on SCR were in the same direction as in the RT measure.

**Figure 8:**
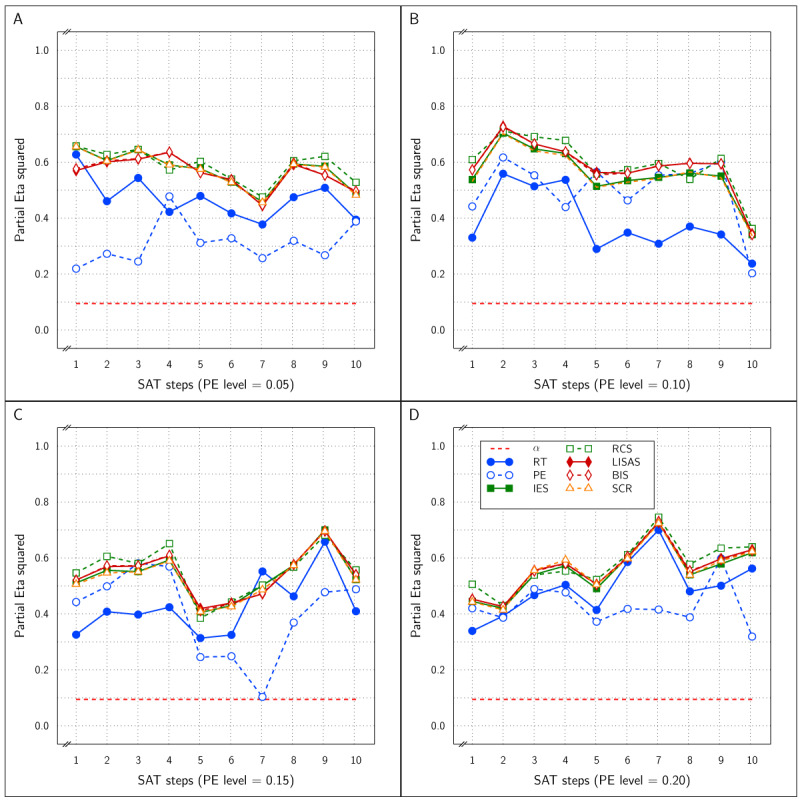
Estimated effect size (\eta _p^2) of the Test effect as a function of the variation in PE (panels A to D) and in SAT size for RT and PE and for the five combined measures in Study 3.

The Test effect is shown in ***Figure 8*** and was significant for all measures at all levels of SAT strength at all four PE levels. Nevertheless, the effect size was variable over the samples. Overall, the effect sizes of the integrated measures were mutually comparable and higher than the RT and PE effect sizes, even though occasionally either the RT or the PE effect attained the same level as the integrated measures, with on average larger effect sizes in RT than in PE.

**Figure 9:**
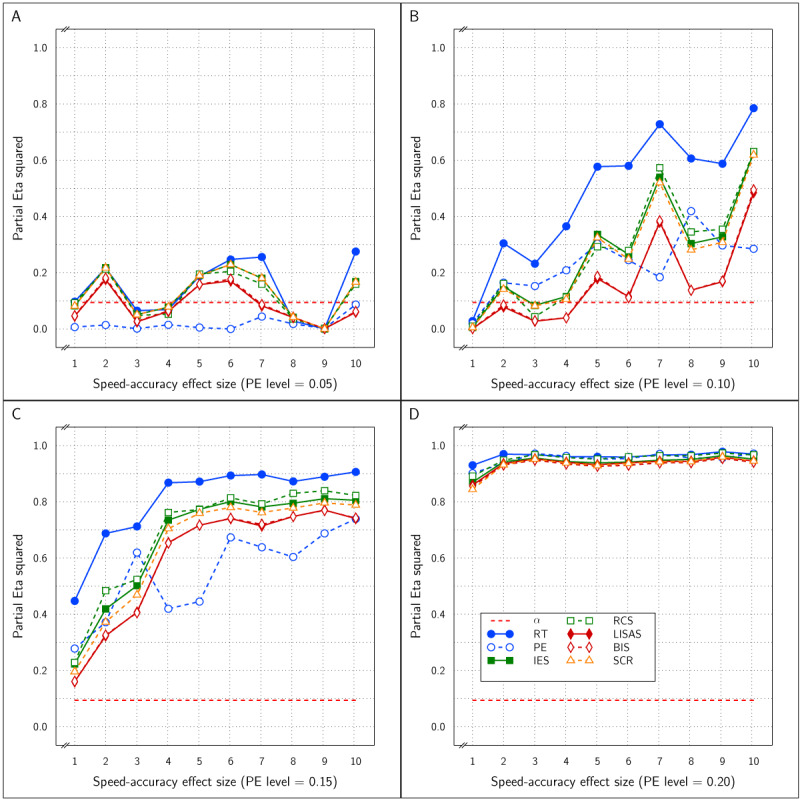
Estimated effect size (\eta _p^2) of SAT Settings as a function of PE level and SAT strength for RT and PE and the five combined measures in Study 3. The dashed line (labeled *α*) represents the significance threshold for these data.

***Figure 9*** displays the SAT size effect as detected by all the measures as a function of PE level and SAT size. The effect sizes increased with PE level and with the strength of the SAT modulation, even though the effect size was strongly variable over SAT modulation strength. At all four PE levels, the RT effect size was dominant. The effect sizes were quite variable at the lower PE levels and did not attain significance in all samples. At the PE level of 0.05, the PE effect size was never significant, while the effects of RT, IES, RCS and SCR were significant in half of the samples and the effect of LISAS and BIS attained significance in 3 of the 10 samples (SAT strengths). At PE level of 0.10, the RT and PE effects were significant in 9 out of the 10 samples, the effects of IES, RCS and SCR attained significance in 8 out of the 10 samples, whereas LISAS and BIS were significant in 5 out of 10 samples. At PE levels 0.15 and 0.20 all the measures were significant all the time.

With the present basic design in which the SAT factor consisted of a contrast between no SAT variation at all versus SAT modulation present, an interaction of SAT and Test may be expected, as the SAT modulation is expected to change the size of the Test effect. Because the Test effect implemented in this Study is the same as that defined in Study 2, it is expected to be detected as well when SAT modulation occurs as when it does not occur. The interaction was never significant at PE levels of 0.05 and 0.10, but at level 0.15, the interaction was significant for RT at SAT sizes 8–10, for PE only at SAT size 10, and for the integrated measures, it was significant at SAT size 8 and 9 only. In contrast, at PE level 0.20, the interaction was significant for all the measures at all SAT sizes, except for PE which did not attain significance at SAT size 1 (*p* = 0.89, \eta _p^2 < .01).

This simulation based on error-driven (reactive) SAT modulations showed a pattern of findings that highly varied as a function of overall PE level. In samples with a high error likelihood, all measures are clearly sensitive to the SAT modulation, even if the size of the modulation is very small. However, when error likelihood is smaller, the larger the SAT modulation, the larger the probability it is detected, and this was the case for all the measures studied in the present simulations.

## Study 4: Discontinuous Speed-Accuracy Trade-off

In contrast to DDM and other sequential-sampling models, speed-accuracy modulation has also been conceived as a discontinuous process, which consists of an alternation between slow and accurate responding on the one hand and fast guessing responses on the other hand (Ollman, 1966; Yellott, 1971). However, this type of modeling has been found to suffer from many shortcomings and it typically fails to provide an appropriate explanation of SAT (Heitz, 2014). Nevertheless, one particular discontinuous (two-phase) model seems to be able to account for a few findings that are difficult to explain by sequential-accumulation models, namely the model proposed by Dutilh et al. (2011). Although, this model may still not be able to account for major findings regarding SAT, these authors have shown that for larger error rates the model yields a better account of the findings than continuous diffusion-like models and, in particular, they have shown that it can account for the presence of bimodal response distributions when the range of errors is large. A basic assumption of such discontinuous models is that errors are considered to be rare under slow processing, but fast responses, on the contrary, are considered to be fast guesses because those responses occur without proper processing of stimulus information. Consequently, with binary choices, such fast responses are expected to be correct on only 50% of the trials. In order to achieve a level of 75% correct responses, this means that on half of the trials a fast guess may occur (resulting in 25% correct trials) and that on the other half of the trials, slow correct responses are produced resulting in another 50% correct responses. Gradual or continuous modulation is nevertheless considered to be possible in the slow mode, but only for percentages up to 5–10% errors.

The present study was performed using a discontinuous model in which the fast responses were guesses achieved without any processing of inputs (50% correct), and the slow processing mode was modeled as a standard diffusion process as applied in Study 2.

### Method

Fast guessing responses had an actual decision time of zero and a non-decision time of 0.25 s in a gaussian distribution with a standard deviation of 0.04 s. Parameters for the diffusion component of the two-phase model had the same values as those used in Study 2, corresponding to the two PE levels of 05 and 0.10. Similar to Study 2, a fixed non-decision time of 0.3 s was added.

A 2 (PE levels) × 5 (SAT strength steps) design was used for the present study with each cell based on a sample with a 2 (Test: Control vs. Experimental) × 5 (SAT Targets) repeated measures design. The SAT Targets in this basic design represented the instruction to achieve a particular target level of correct responding varying in steps of 5% from 75% to 95%. Over the simulations, PE level variation was limited to the range [0.05–0.10] and thus had only these two values. The fast guessing mode had no variations, but in the slow response mode (diffusion part of the model), SAT level was varied in 5 steps between 0.001 and 0.005, in such a way that in the 90% target condition the response boundary was decreased (leading to faster and more incorrect responses) and in the 95% target condition the response boundary was increased to achieve slower and more correct responses. Clearly, this variation concerned only a limited part of the SAT effect present in these simulations, as the targeted proportion of errors was varied between 0.25 and 0.05.

### Results

**Figure 10:**
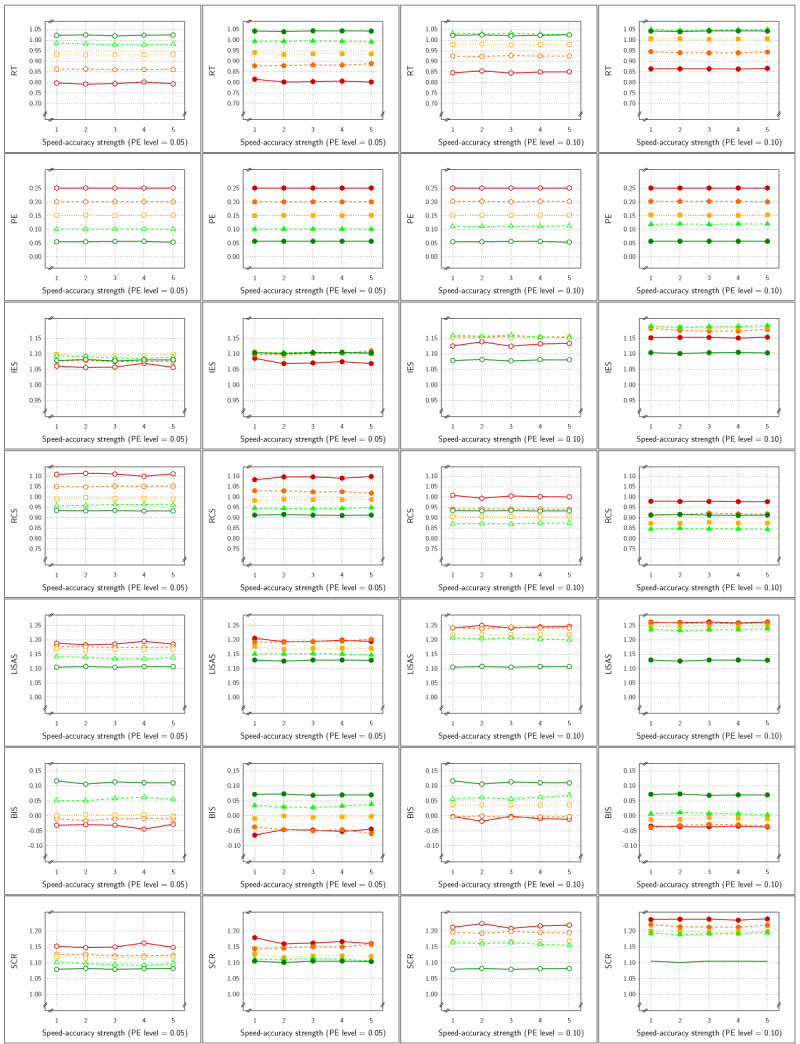
Sample Means in Study 4 as a function of Test × SAT Targets × SAT size (x-axis in each panel) × PE level. Each row of panels shows the means of a single dependent variable with respect to PE level × Test, such that the two panels on the left show the control and the experimental condition means at PE level 0.05, and the two panels on the right show the control and experimental condition means at PE level 0.10. Legend: Open circles for control condition and closed circles for experimental condition; red solid lines for 75%, orange dashed lines for 80%, yellow dotted lines for 85%, green dashed lines for 90%, and dark green solid lines for 95% target.

The means of the Test × SAT Targets cells are displayed in ***Figure 10*** as a function of average PE level and SAT strength steps. At each PE level, the means are shown in separate panels for the Control condition (left) and the Experimental condition (right), so that the two leftmost columns present the means at PE level 0.05, and the two rightmost columns present the means at PE level 0.10. The effect of targeting different proportions of errors is most clearly visible in the second row of panels which show the PE means. These panels show that the targets were achieved: each condition shows approximately the imposed proportion of errors (0.25 for the 75% condition; 0.20 for the 80% condition, etc.). In the PE measure, no clear visual effects of SAT strength were present; only in the panels corresponding to PE level 0.10, the curves for the 10 and 5% targets (which rely mostly on the diffusion phase in the model) showed small deviations from parallelism. All the other measures were more strongly affected by the SAT strength in the diffusion phase: while the curves that are strongly dependent on guessing (range of red to yellow lines) did not vary with SAT strength, the other (green) curves clearly did. Note that in this study like in Study 1, the SAT effects on the SCR measure are reversed with respect to the effects on RT.

**Figure 11:**
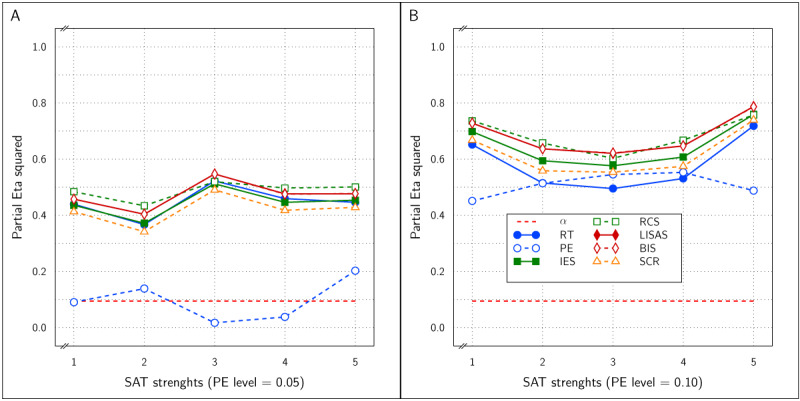
Estimated effect size (\eta _p^2) of the Test effect as a function of the variation in PE (panels A and B) and in SAT size for RT and PE and for the five combined measures in Study 4.

At PE level 0.10, all dependent variables reliably detected the Test effect with \eta _p^2 between 0.5 and 0.8, as can be inferred from panel B of ***Figure 11***. Noteworthy, the integrated measures achieved larger effect sizes than RT and PE. At PE level 0.05 (panel A of ***Figure 11***), the results were similar, but strikingly the PE effect sizes hovered around the significance level, while all the other measures attained \eta _p^2 between 0.35 and 0.55 without clear distinctions between these measures.

**Figure 12:**
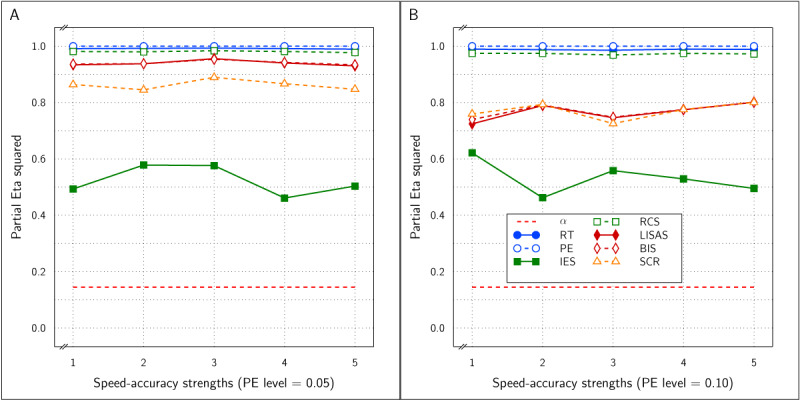
Estimated effect size (\eta _p^2) of SAT Settings as a function of PE level and SAT strength for RT and PE and the five combined measures in Study 4. The dashed line (labeled *α*) represents the significance threshold for these data.

***Figure 12*** shows the effect size of the SAT Target factor in all samples of the present study as a function of PE level and SAT size. All measures were sensitive to the SAT Target effect at both PE levels and at all SAT strength levels. In fact, for RT, PE, and IES, the effect sizes were near the maximal value of 1.0, while LISAS, BIS and RCS effects were lower, but still quite large. Remarkably, the RCS effects were the lowest at both PE levels.

Although in some of the samples, some of the measures revealed an interaction of Test and SAT modulation, ***Figure 10*** clearly shows that in every measure, the corresponding SAT target curves in the control and experimental conditions are different. Taken together with the strong overall Test effect, the present findings suggest that the Test effect is present independently of SAT modulation effects.

Clearly, even if proactive SAT modulation conforms to a discontinuous trade-off model, not only the primary measures RT and PE, but all combined measures in the present study proved to be sensitive to these modulations, while at the same time being efficient at detecting the Test effect.

## General Discussion

In four studies proactive and reactive speed-accuracy settings were studied in mixed conditions repeated measures designs. Each study used a Test (experimental variable) × SAT Settings repeated measures design that was implemented with two (Study 4) or four (Studies 1–3) variations in the average PE level and five (Study 4) or ten (Studies 1–3) levels of strength in the SAT factor. The SAT Settings were either based on proactive SAT modulations varying the settings from speed stress over neutral to accuracy stress (Studies 1 and 2), proactive SAT instructions to achieve a predefined percentage correct target (Study 4) or reactive SAT modulation (Study 3). In these simulations with proactive modulation, no reactive SAT modulations occurred in order to clearly separate the effects of proactive and reactive modulations.

### Summary of the Findings

In all four studies, RT and PE detected the effects introduced by the variations in the basic design (Test and SAT Settings) and the variations over the samples (PE level and SAT strength), but the strength of the effects varied over the studies. That the basic measures detected the effects is important as it is a prerequesite to draw reliable conclusions about the measures that integrate RT and PE, and it is also needed to allow a study of the effects of these same factors on the integrated measures.

The five integrated measures included in the present studies can be partitioned into two groups.

1. On the one hand, IES, RCS and SCR are the result of taking the ratio of RT and PC (converse of PE) either with RT (IES) or PC (RCS) in the numerator of the fraction, or by means of a weighted mixture of correct and incorrect RTs (SCR).
2. LISAS and BIS on the other hand are also very similar to each other as explained in Appendix A and are based on a weighted sum of RT and PE.

In all four studies very similar results were obtained for IES, RCS and SCR. First, detection of the effect of the experimental variable (Test effect) by these three measures was indistinguishable and almost identical in all four studies. Second, regarding the size of the SAT strength, except for Study 4, the effects detected by these measures were similar and almost indistinguishable: in ***Figure 3*** (Study 1) and in panels A and B of ***Figure 9*** (Study 3) the curves of IES, RCS and SCR are very close together, while in ***Figure 6*** (Study 2) and in panels C and D of ***Figure 9*** (Study 3) the curves for these three measures are grouped with some of the other measures, The only exception is in Study 4, where RCS appeared to be less sensitive to the size of the SAT factor than the other measures (lower curve for RCS in ***Figure 12***). The sensitivity to SAT strength was moderated by PE level in some of the studies, but the direction of this moderation differed over the studies.

Contrary to what may be expected from Liesefeld and Janczyk (2019), the results obtained with LISAS and BIS were barely distinguishable. First with respect to detection of the effect of experimental conditions, LISAS and BIS were both very efficient at a level similar to IES, RCS and SCR in all the studies. Second, variability in findings was quite large for sensitivity to detect SAT effects: both LISAS and BIS failed to detect the SAT effect in Study 1 which used a linear and balanced modulation of RT and PE. In all the other studies, LISAS and BIS reliably detected the SAT modulation effect at high levels, but very frequently with lower effect sizes than the other integrated measures. Nevertheless, in Studies 2–4, the SAT effect was detected reliably by LISAS and BIS.

These observations trigger the question why Liesefeld and Janczyk (2019) found larger differences between BIS and LISAS than the present simulations. One possible reason for this difference is quite likely due to the usage of between-subject designs in the Liesefeld-Janczyk paper. In between-subjects designs, the standard deviations used by LISAS are estimated from one single condition, so that in these circumstances no unbiased estimates are available. In fact, LISAS was explicitly defined and presented in a within-subjects context so that the pooled standard deviations over al conditions by the same subject can be used (Vandierendonck, 2017). Therefore, when used in a between-subjects context, LISAS can only be calculated when proper estimates of the standard deviations of RT and PE are used; hence, in a between-subjects design such estimates should be based on the sample standard deviations of RT and PE. In the present simulations, only repeated measures designs were used, so that proper estimates of the standard deviations of RT and PE were available.

According to some models, SAT is the result of a mixture of fast guesses (with error probability of 0.5 for binary response choices) and slow stimulus controlled responses. As shown by Yellott (1971) on the basis of these models, average latency of these stimulus controlled responses can be estimated by means of the SCR measure (Eq 10). Yellott’s own experimental results suggest that these estimates are quite stable, and did not vary much with the imposed response deadlines. In the present simulations, which are not based on such simple models, the measure was as sensitive to SAT variations as the other integrated speed-accuracy measures in all four studies. In Studies 1 and 4, the SAT effects on SCR were reversed, so that the faster responses with higher error rates in the speeded condition resulted in a higher SCR, while the slower more correct responses in the accuracy stress condition resulted in a lower SCR. This looks like an overcorrection of the SAT adaptation. In Study 4, which was based on a mixed model with fast guessing and SAT modulation on the slower responses, the SCR scores were sensitive to the SAT effect. This runs against expectations based on a fast guessing model. In short, the SCR scores did not behave as could be expected, and the present findings therefore confirm the misgivings that have been formulated with respect to discontinous SAT models (e.g., Heitz, 2014, Stafford et al., 2020).

With respect to the situations used in the present simulations, the question may also be raised whether realistic conditions were implemented. Actually, two different issue of concern must be addressed in this respect. First, to what extent is the usage of a design that crosses a task factor with a SAT settings factor realistic? Second, were realistic trade-off settings applied?

The first question concerns the design used. All four simulation studies were based on a design which crossed an SAT settings factor with the factor of main interest (Test). This design, formalised in the structural model of Eq (11), was selected because it is an appropriate design to answer the research questions under consideration, namely

1. Is it indeed the case that some or maybe all integrated measures sufficiently compensate modulations in RT and PE so that they can be considered to be insensitive to SAT variations?
2. Do the integrated measures reliably detect the Test effect irrespective of the SAT conditions?

Although it is clear that this design is appropriate to answer these questions, this design does not provide a general tool for any research concern involving integrated measures. In fact, if the results of the present simulations are considered to be valid, there is no need for other studies to explicitly include SAT manipulations, as it has already been shown that the integrated measures tested in the present article are sensitive to SAT modulations and do not sufficiently compensate deviations between the two basic measures of speed and accuracy. It suffices, however, to use a mixed conditions repeated measures design to ensure that the SAT variations that are bound to occur during an experimental sessions are balanced over the task conditions so that the task effects studied are properly detected.

The second question concerns the realism in the modelling of SAT. Study 1 used a quite strongly constrained model of the trade-off and obtained results that cannot be generalised beyond these specific conditions. In the other studies, the modelling was based on the drift diffusion model (Ratcliff, 1978), and this model is broadly believed to provide an adequate representation of SAT (Stafford et al., 2020).

The Test and SAT Settings factors did not interact in Study 1, but interactions were present in the other studies. No predictions were attempted about the size and the pattern of these interactions, because this is mathematically not straightforward. Moreover, in the simulations based on the drift diffusion model, interactions are produced by the operations defined in the model. However, it is important to note that no cross-over interactions were observed. The presence of such interactions would cast doubt on the validity of the observed Test effects, as their size and direction would depend on the specific combination of Test and SAT setting conditions. For all studies it was clear that all measures reliably detected the effect of the experimental variable (Test) at all levels of the SAT settings, and the SAT Settings effect was detected reliably in both experimental conditions.

In summary, it can be said that

1. The basic measures as well as all the integrated measures reliably detected the effect of the experimental variable (Test) irrespective of the size of the strength of the SAT modulations and the level of PE.
2. Except for the insensitivity to the proactive linear balanced SAT modulation in Study 1, LISAS and BIS were sensitive to all SAT modulations in all the other studies.
3. IES, RCS and SCR were sensitive to all SAT modulations even when the size of the effect was small.
4. Whether Test and SAT Settings interacted or not, in all studies, the Test effect was reliably detected at all SAT Settings and the SAT Settings effect was reliably detected in both experimental conditions.

### Implications of these Findings

The present simulations show that *no single measure is completely insensitive to SAT*. Not only the direct victims of SAT modulations, RT and PE, but also IES, RCS and SCR reliably detected the SAT effects and did so even more reliably as the strength of the modulation increased, and this was confirmed in all four studies (except, Study 4 for RCS). The same goes for LISAS and BIS, except that these two measures, as expected on the basis of their definition, are insensitive to linearly balanced SAT effects as implemented in Study 1. That IES and RCS did not reveal any insensitivity to SAT effects is probably a consequence of not including a study which defined SAT settings with proportional modulations as explained in the introduction when these measures were introduced. Nevertheless, the results obtained in the simulations reported in this article inevitably lead to the conclusion that none of the integrated speed-accuracy measures presently available can be trusted to be unconditionally insensitive to SAT. Therefore, extending the advice formulated by Townsend and Ashby (1983) *these integrated measures should not be used to neutralise or to circumvent SAT effects*.

More importantly, the present simulations also show that there is in fact no need to neutralise or to circumvent SAT effects, as the simulations also showed that in the context of mixed conditions repeated measures designs, experimental effects are reliably detected irrespective of the presence and the size of the SAT modulations. In fact, in all simulation studies reported here, all the integrated measures reliably detected the effect of the experimental variable irrespective of whether SAT was present (e.g., Study 3), or irrespective of which SAT Settings were used (all studies), or whether the experimental variable did or did not interact with the SAT variation. In other words, within the constraints of the present studies, detection of the experimental effect was robust in the face of the presence of different SAT settings. No doubt, the imposed constraints are very important, namely the usage of a mixed conditions repeated measures design, that means a design with the experimental conditions occurring in a random or pseudo-random sequence so as to prohibit triggering of condition-based SAT Settings, and to dilute the SAT effects over the experimental conditions. However, as argued in the introduction, if it is suspected that SAT modulations may be present, and SAT modulations are not directly triggered (by imposing response deadlines, by instruction, by incentives, etc.), it is imperative to use (a) repeated measures designs, (b) with mixed conditions, so that the experimental effects under study occur within subjects and the possible trade-offs have their effects spread over the conditions. With between-subjects studies, both within-subject SAT compensations and between-subject speed-accuracy compensations may affect the outcomes and threaten the validity of the study. Hence, by using a mixed condition repeated measures design, the present simulation studies support the conclusion that when a design with these properties is used, detection of an independent or orthogonally varied experimental variable is not hindered by the presence of SAT effects, and this is true for *all* the measures, including RT and PE.

Linked to this implication, it also follows that between-subject designs where by definition the conditions have to be blocked by subject and/or by session should be avoided, because in such designs, condition-wise SAT settings may form a confounding factor. Adepts of sequential-sampling SAT models may still hold to their advise to continue to use such designs with estimation of the diffusion model parameters to understand how the response decision was made (e.g., Stafford et al., 2020). As already explained in the introduction, however useful such an advise may be in some research contexts, there are probably a number of research questions in which it would be better to simply use a repeated measures design with a randomized task sequence (see also Wagenmakers et al., 2007). Many researchers prefer to work with variables that express directly observable phenomena rather than with theoretical variables that are linked to the observables by rather complex mathematical formulae. Moreover, it is also the case that many psychological phenomena of interest are situated in the non-decision time; these effects may be obscured by the presence of SAT, but are not dependent on it. Therefore, it seems to be a useful strategy to try to neutralise SAT by spreading it over conditions of interest.

Finally, as to the decision which integrated measures can best be used in order to be secured against SAT modulations, it seems that none of the five measures is more advantageous than the other measures. That being said, there is no reason to assume that these measure are equally efficient in detecting the effect of a task manipulation. From previous research it is known that one should be careful in the choice of an integrated measure. For example, one should be careful in using IES and RCS when PE levels are very variable among participants in the study and may occasionally be very high. Moreover, these measures seem to be less efficient than LISAS and BIS (Vandierendonck, 2017, 2018) and can lead to surprises (Bruyer & Brysbaert, 2011; Vandierendonck, 2018). Furthermore, the present simulations show that there is barely any difference between the results obtained with LISAS and BIS. However, in terms of transparency and independence from sample parameters, LISAS has a number of advantages over BIS, as it is calculated independently from the score of other subjects in the sample and it yields a measure that can be interpreted as an RT corrected for errors.

## Conclusion

Although the presence of SAT often creates nuisance with respect to the interpretation of findings, the present paper shows that all measures used are extremely sensitive to SAT settings. Therefore, these measures are not well suited to directly neutralise SAT. However, the present article also shows that when a mixed conditions repeated measures design is used, the variable of interest is not confounded by SAT effects. With such a design, as well for response time, proportion of errors as for the scores that are a combination of these two measures the effects of interest are reliably detected notwithstanding the presence of speed-accuracy trade-offs.

## Additional files

The additional files for this article can be found as follows:

10.5334/joc.154.s1 Appendix A. LISAS-BIS Relationship.

10.5334/joc.154.s2 Appendix B. Example.

## Footnotes

This measure was not included in the present simulations because it largely depends on some arbitrary choices that have to be made about the values to be assigned to the parameters RT*_min_* and A. An exploratory application to the simulation data of Study 2 indicated that under the assumptions made for the parameter values, the performance of this measure was comparable to that of the other integrated measures in the present study.

The simulation results of all four studies reported in this paper are available at *https://doi.org/10.5281/zenodo.4593016*.

Note that non-decision time in the DDM represents the time that is not spent in response selection, but is taken up in perception of the stimuli and motor execution of the response, and hence is unaffected by the other DDM parameters.

In Study 1, samples of 100 statistical subjects were used to ensure that any insensitivity to SAT would have enough power to accept the null hypothesis. In the present study, no such precaution is required, and therefore a number of 40 statistical subjects was large enough to guarantee sufficient power as it would be required in a typical experiment.
