# Correction: On the Utility of Integrated Speed-Accuracy Measures when Speed-Accuracy Trade-Off is Present

**DOI:** 10.5334/joc.192

**Published:** 2021-11-10

**Authors:** André Vandierendonck

**Affiliations:** 1 Department of Experimental Psychology, Ghent University, Henri Dunantlaan 2, B-9000 Gent, BE

**Keywords:** Integrated Speed-Accuracy Measures, Speed-Accuracy Trade-Off, Drift-Diffusion Model

## Abstract

This article details a correction to: Vandierendonck, A. (2021). On the Utility of Integrated Speed-Accuracy Measures when Speed-Accuracy Trade-off is Present. *Journal of Cognition, 4*(1), 22. DOI: *https://doi.org/10.5334/joc.154*

## Correction

It came to the author’s attention that in the simulations presented by Vandierendonck (2021) an incorrect formula was used in the calculations of the integrated speed-accuracy measure BIS (Liesefeld & Janczyk, 2019). The pooled standard deviation over trials, conditions, and subjects was used instead of the standard deviation over subjects after aggregating trial and condition data per subject and for the speed data only correct instead of all RTs were used. A recalculation shows that in all four simulation studies the correct values tended to differ from the ones in the publication. In the present article, the correct outcomes for the BIS measure in each study are reported. The data and the scripts for these calculations are available at *https://doi.org/10.5281/zenodo.5493844*.

## Study 1: Balanced Speed and Accuracy Effects

The correct BIS means of the cells of the design are shown in ***Figure 1*** as a function of percentage of errors (PE level, the four panels in the figure) and SAT size (the x-axis in each panel); within each panel the means are shown as a function of the 2 (Test condition) × 3 (directions of SAT) on the y-axis.

**Figure 1:**
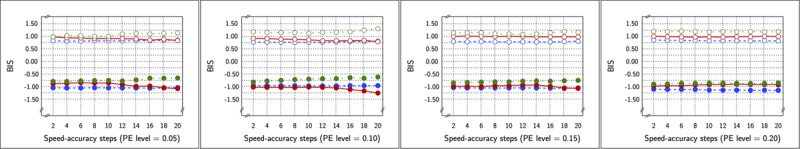
Sample Means in Study 1 as a function of Test × SAT Settings × SAT size (x-axis in each panel) × PE level (panels from left to right) for correct BIS. The curves within each panel show the cell means of the Test × SAT Settings combinations. Legend: open circles for control condition and closed circles for experimental condition; red solid lines for speed stress, blue dashes for neutral SAT, and green dotted lines for accuracy stress.

The Test effect on the corrected BIS measures (henceforth, BIS_c_) was significant in all 40 replications (4 PE levels × 10 SAT size steps) and the \eta _p^2 (partial eta-squared) values ranged between 0.613 and 0.740 (compared to 0.652–0.712 for the original values which also attained significance in all 40 cases). The results of BIS_c_ and all the other measures in the study are shown in ***Figure 2*** as a function of SAT size in four panels, one per PE level.

**Figure 2:**
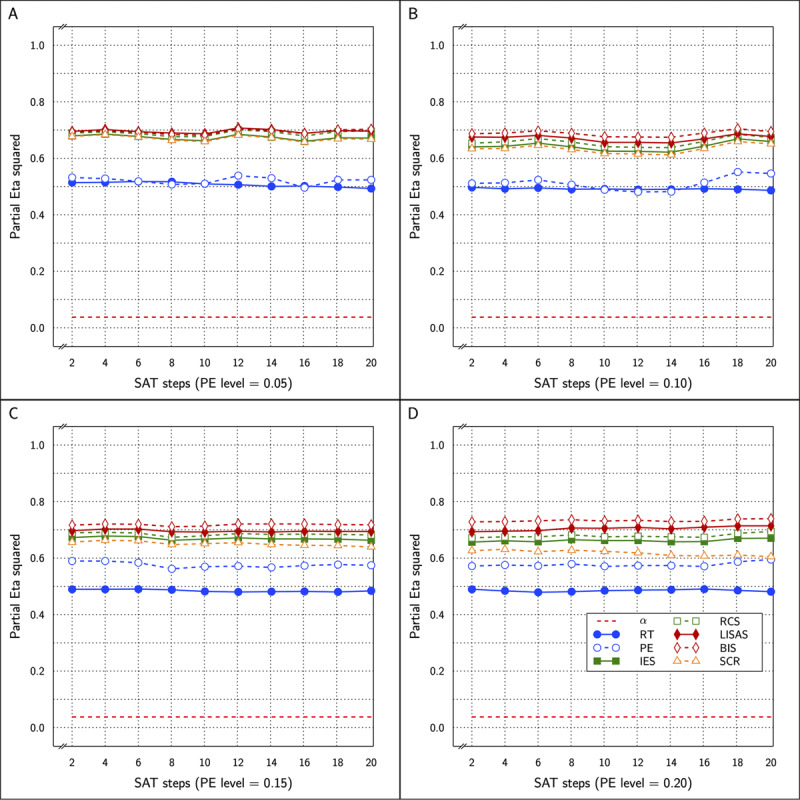
Estimated effect size (η_p_^2^) of the Test effect as a function of the variation in PE (panels **A** to **D**) and in SAT size for RT and PE for the five combined measures in Study 1.

Similarly, the SAT effect on BIS_c_ was significant in 5 of the 40 replications with \eta _p^2 between 0.014 and 0.131 (originally 0 of 40 replications were significant and \eta _p^2 varied between 0.016 and 0.057. ***Figure 3*** shows these findings for all the measures per PE level and SAT size.

**Figure 3:**
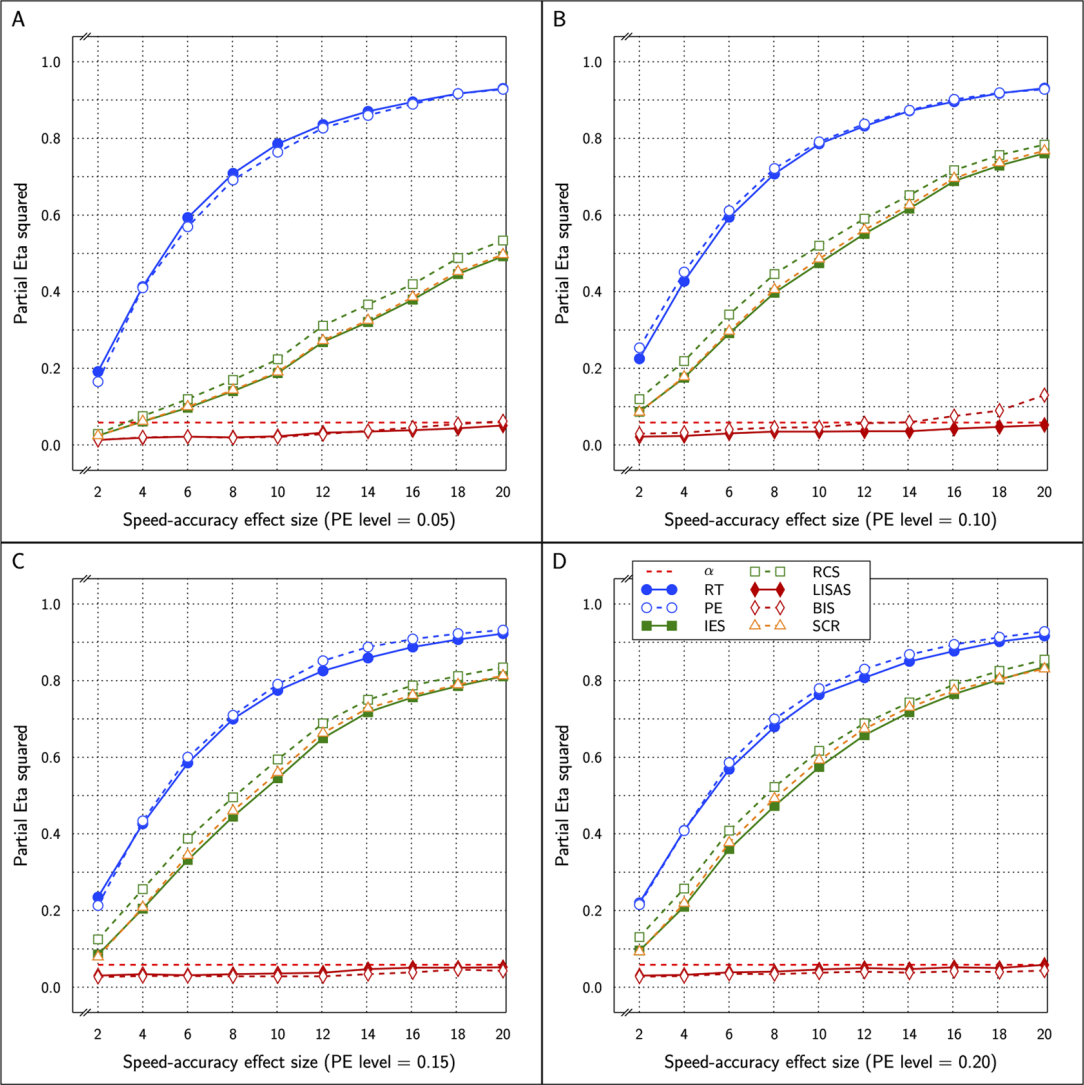
Estimated effect size (η_p_^2^) of SAT Settings as a function of PE level and SAT strength for RT and PE and the five combined measures in Study 1. The dashed line (labeled α) represents the significance threshold for these data.

## Study 2: SAT Effects based on the Drift-Diffusion Model

Recalculation of BIS_c_ in the second study revealed also small differences compared to the incorrect calculations. The means are displayed in ***Figure 4***.

**Figure 4:**
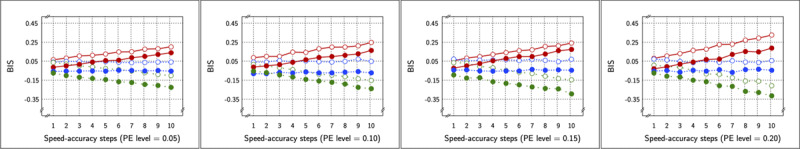
Sample Means in Study 2 as a function of Test × SAT Settings × SAT size (x-axis in each panel) × PE level (panels from left to right) for BIS_c_. The curves within each panel show the cell means of the Test × SAT Settings combinations. Legend: open circles for control condition and closed circles for experimental condition; red solid lines for speed stress, blue dashes for neutral SAT, and green dotted lines for accuracy stress.

As with the original results, the Test effect was significant in all 40 replications (PE level × SAT size) with \eta _p^2 varying from 0.518 to 0.739 (0.518 to 0.712 in the original results). ***Figure 5*** displays the test effect for all measures in the study as a function of PE level (4 panels) and SAT size within each panel.

**Figure 5:**
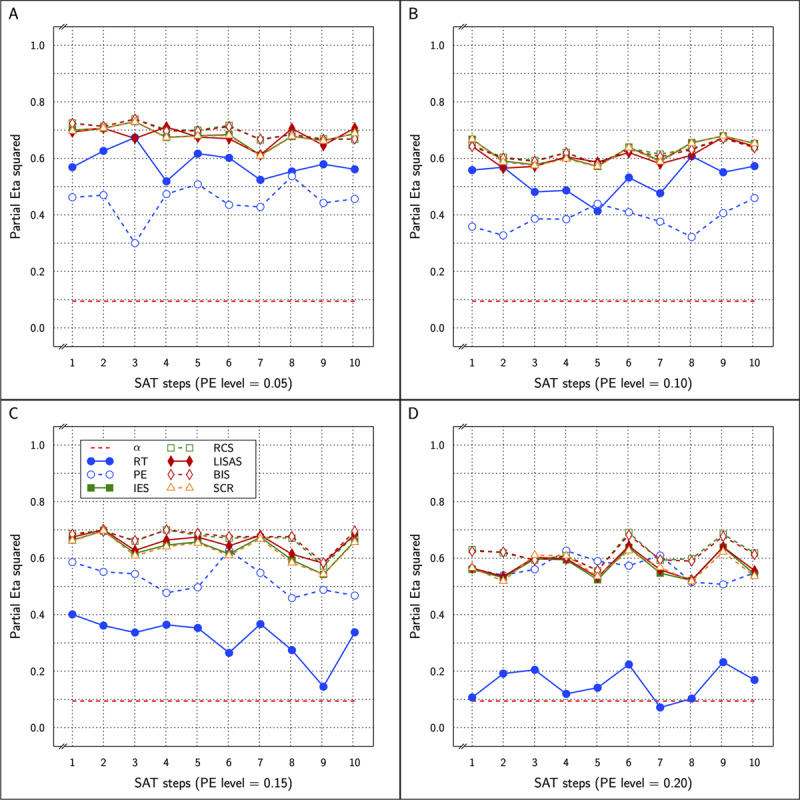
Estimated effect size (η_p_^2^) of the Test effect as a function of the variation in PE (panels **A** to **D**) and in SAT size for RT and PE for the five combined measures in Study 2.

Whereas the SAT effect was significant in 39 out of 40 replications in the original calculations, the corrected calculations yielded 40 significant SAT effect sizes with \eta _p^2 ranging from 0.152 to 0.956 (0.141 to 0.955 for the original outcomes). The correct effect sizes of all the measures in the study are displayed in ***Figure 6***.

**Figure 6:**
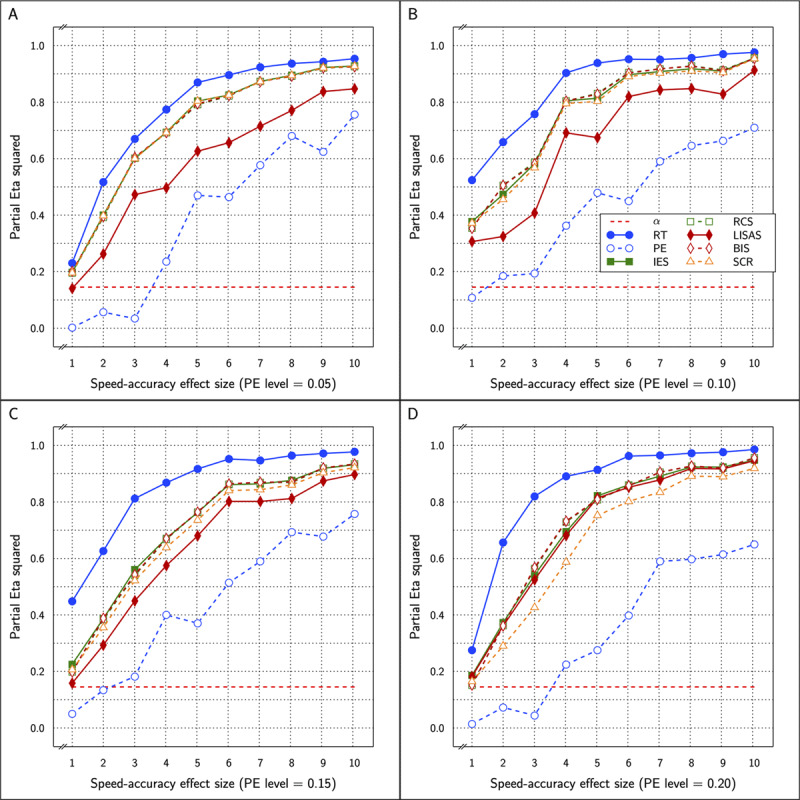
Estimated effect size (η_p_^2^) of SAT Settings as a function of PE level and SAT strength for RT and PE and the five combined measures in Study 2. The dashed line (labeled α) represents the significance threshold for these data.

## Study 3: Reactive Speed-Accuracy Modulations

In the third study, the basic design was different and crossed a Test effect with conditions without and conditions with SAT. This design was also replicated over 4 PE levels and 10 SAT sizes. The means for BIS_c_ are displayed in ***Figure 7***, one panel per PE level.

**Figure 7:**
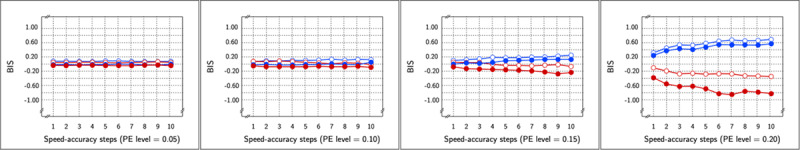
Sample Means in Study 3 as a function of Test × SAT Settings × SAT size (x-axis in each panel) × PE level (panels from left to right) for correct BIS. The curves within each panel show the cell means of the Test × SAT Settings combinations. Legend: Blue for SAT-absent conditions, red for SAT-present conditions; open circles for control condition, closed circles for experimental condition.

As in Studies 1 and 2, the Test effect on the correct and the incorrect calculations were very similar: in both the test effect was significant in all 40 replications with \eta _p^2 varying from 0.364 to 0.739 in the correct calculations compared to a range between 0.341 and 0.731 in the incorrect analyses. ***Figure 8*** shows the correct outcomes for all the measures in the study.

**Figure 8:**
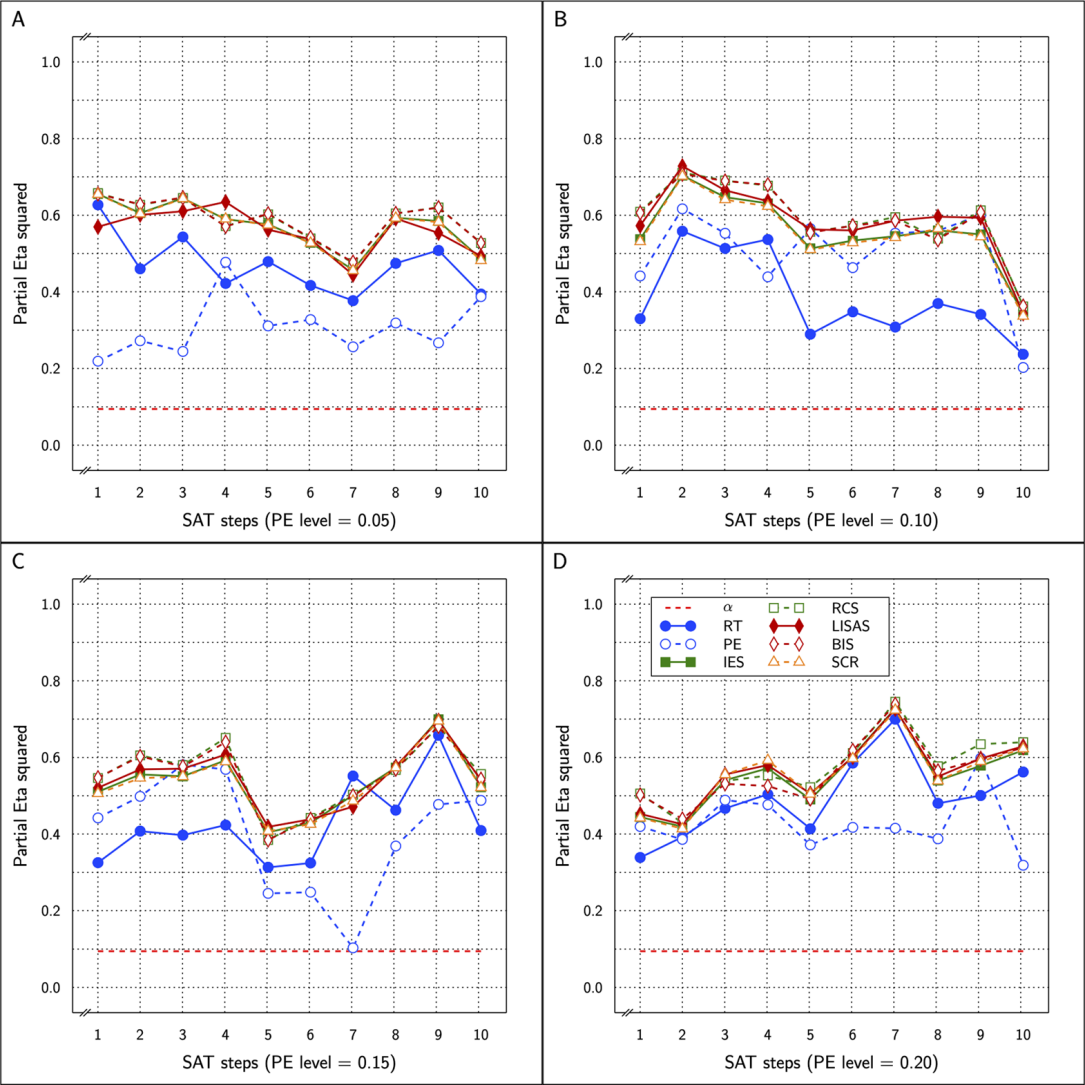
Estimated effect size (η_p_^2^) of the Test effect as a function of the variation in PE (panels **A** to **D**) and in SAT size for RT and PE for the five combined measures in Study 3.

The SAT effect of BIS_c_ was significant in 31 of the 40 replications with \eta _p^2 varying between 0.001 and 0.962 which is very similar to the outcomes of the incorrect values which were significant in 29 out of 40 cases and \eta _p^2 varying between 0.001 and 0.960. The correct values are shown with the outcomes of the other measures in ***Figure 9***.

**Figure 9:**
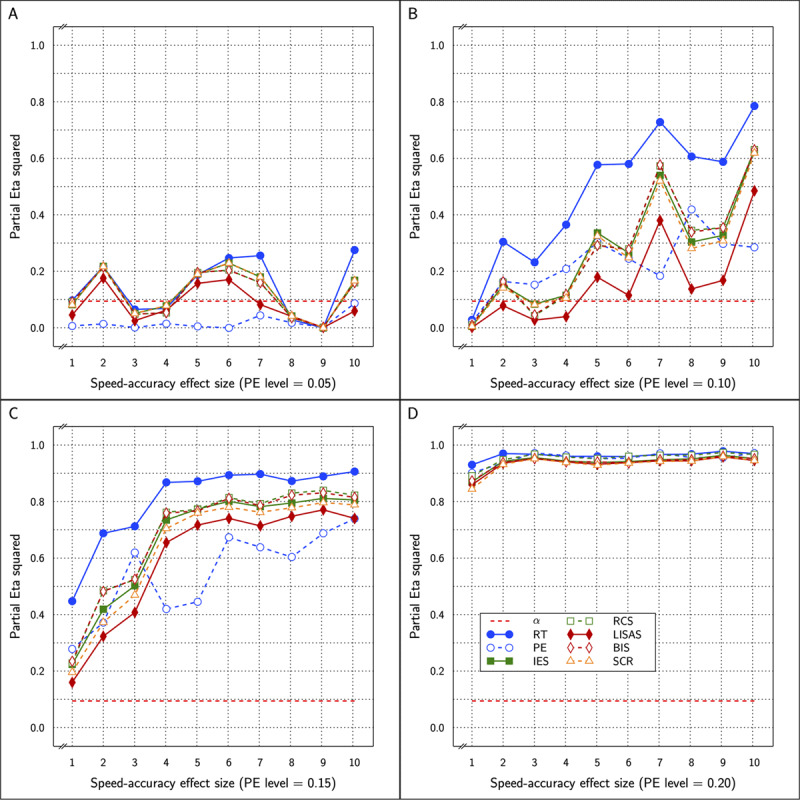
Estimated effect size (η_p_^2^) of SAT Settings as a function of PE level and SAT strength for RT and PE and the five combined measures in Study 3. The dashed line (labeled α) represents the significance threshold for these data.

## Study 4: Discontinuous Speed-Accuracy Trade-off

In this study a discontinuous model of speed-accuracy trade-off was tested by varying the SAT target levels that had to be achieved. The basic 2 (Test) × 5 (Targets) design was replicated in 2 PE levels × 5 Target size steps. The BIS_c_ means are displayed in ***Figure 10***.

**Figure 10:**
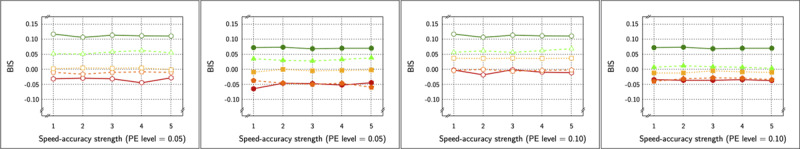
Sample Means in Study 4 as a function of Test × SAT Targets × SAT size (x-axis in each panel) × PE level. The row of panels shows the BIS_c_ means with respect to PE level × Test, such that the two panels on the left show the control and the experimental condition means at PE level 0.05, and the two panels on the right show the control and experimental condition means at PE level 0.10. Legend: open circles for control condition and closed circles for experimental condition; red solid lines for 75%, orange dashed lines for 80%, yellow dotted lines for 85%, green dashed lines for 90%, and dark green solid lines for 95% target.

The Test effect on the correct calculations was significant in 10 out of 10 replications with \eta _p^2 values between 0.495 and 0.800 which mimicked the outcomes of the original calculations which were also significant in 10 cases with \eta _p^2 between 0.404 and 0.787. The correct Test effect \eta _p^2 values are displayed in ***Figure 11***.

**Figure 11:**
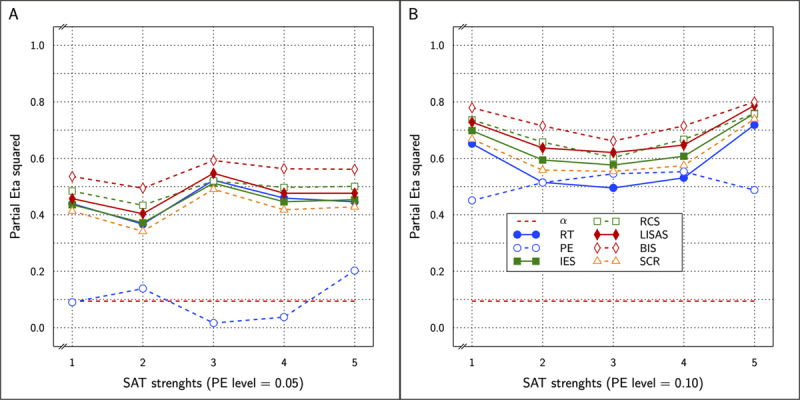
Estimated effect size (η_p_^2^) of the Test effect as a function of the variation in PE (panels **A** and **B**) and in SAT size for RT and PE and for the five combined measures in Study 4.

Similarly, for the SAT effects, the correct calculations were significant in all 10 cases with \eta _p^2 values between 0.755 and 0.901 compared to values between 0.726 and 0.943 for the incorrect calculations. The correct results are shown in ***Figure 12***.

**Figure 12:**
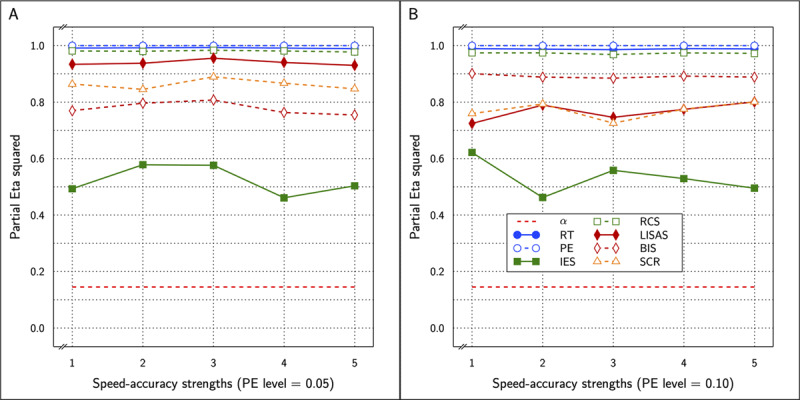
Estimated effect size (η_p_^2^) of SAT Settings as a function of PE level and SAT strength for RT and PE and the five combined measures in Study 4. The dashed line (labeled α) represents the significance threshold for these data.

## Discussion

In all the studies, the recalculations of BIS yielded outcomes that were in the same range as the original calculations with the incorrect formula. Therefore, the discussion of the original outcomes is equally applicable to the recalculated outcomes and the conclusions formulated by Vandierendonck (2021) remain valid.
