# Supplementary material for: On the Utility of Integrated Speed-Accuracy Measures when Speed-Accuracy Trade-off is Present
Source: J Cogn. 2021 Mar 18;4(1):22. doi: 10.5334/joc.154 (PMC7977027; doi:10.5334/joc.154)
Supplement: Appendix A. — LISAS-BIS Relationship. [file joc-4-1-154-s1.pdf]

Appendix A

LISAS-BIS Relationship

This appendix provides a proof that when the BIS score is based on a single-subject sample it is a linear transformation of LISAS, so that in this case the two scores are equivalent. To do so, the notation is simplified: $s_r = S_{\text{RT}_\text{j}}$, $s_e = S_{\text{PE}_\text{j}}$, $z_r = z_{\text{RT}_{ij}}$, and $z_c = z_{\text{PE}_{ij}}$. Furthermore, because PE = (1 - PC), and the underlying binomial distribution, $s_e = s_c$ and $z_e = -z_c$.

The definition of LISAS (4) can be rewritten in standard scores. Starting from the expression for the RT standard scores (eq. 7):

$$z_r = \frac{\text{RT}_{ij} - \overline{\text{RT}}}{s_r} \tag{17}$$

it follows that

$$z_r s_r = \text{RT}_{ij} - \overline{\text{RT}} \tag{18}$$

and

$$\text{RT}_{ij} = z_r s_r + \overline{\text{RT}} \tag{19}$$

Similarly, using equation 6,

$$\text{PE}_{ij} = z_e s_e + \overline{\text{PE}} \tag{20}$$

so that the definition of LISAS can be rewritten by substitution:

$$\text{LISAS}_{ij} = z_r s_r + \overline{\text{RT}} + (z_e s_e + \overline{\text{PE}})\frac{s_r}{s_e} \tag{21}$$

Now, multiply the RT group by $\frac{s_e}{s_e}$ (= 1),

$$\text{LISAS}_{ij} = \frac{s_e}{s_e}[z_r s_r + \overline{\text{RT}}] + (z_e s_e + \overline{\text{PE}})\frac{s_r}{s_e} \tag{22}$$

and distribute s_e in the denominator

$$\text{LISAS}_{ij} = \frac{1}{s_e}[z_r s_e s_r + \overline{\text{RT}} s_e + z_e s_e s_r + \overline{\text{PE}} s_r] \tag{23}$$

Strip off common terms

$$\text{LISAS}_{ij} = [z_r s_r + \overline{\text{RT}} + z_e s_r + \overline{\text{PE}}\frac{s_r}{s_e}] \tag{24}$$

and regroup

$$\text{LISAS}_{ij} = (z_r + z_e)s_r + \overline{\text{RT}} + \overline{\text{PE}}\frac{s_r}{s_e} \tag{25}$$

Consider that $z_r + z_e = z_r - z_c = -\text{BIS}_{ij}$, if follows that

$$\text{LISAS}_{ij} = s_r(\frac{\overline{\text{RT}}}{s_r} + \frac{\overline{\text{PE}}}{s_e} - \text{BIS}_{ij}) \tag{26}$$

and also that

$$\mathrm{BIS}_{ij} = \frac{\mathrm{LISAS}_{ij}}{s_r} - \frac{\overline{\mathrm{RT}}}{s_r} - \frac{\overline{\mathrm{PE}}}{s_e} \tag{27}$$
