# Supplementary material for: On the Utility of Integrated Speed-Accuracy Measures when Speed-Accuracy Trade-off is Present
Source: J Cogn. 2021 Mar 18;4(1):22. doi: 10.5334/joc.154 (PMC7977027; doi:10.5334/joc.154)
Supplement: Appendix B. — Example. [file joc-4-1-154-s2.pdf]

Appendix B

Example

Table B1

Means per cell of the 2 (Test) × 3 (SAT Settings) repeated measures design for each of the measures in Study 1. The table presents the means for overall PE = 0.05 and the defined speed-accuracy effect size of ±20 *ms.*

Measure	Test	Speeded	Neutral	Accurate
RT	C	1023	1046	1065
	E	1033	1056	1076
PE	C	0.237	0.190	0.133
	E	0.263	0.216	0.161
IES	C	1345	1295	1231
	E	1405	1350	1286
RCS	C	0.745	0.775	0.814
	E	0.713	0.743	0.781
LISAS	C	1123	1126	1120
	E	1144	1147	1144
BIS	C	0.068	0.049	0.083
	E	-0.055	-0.071	-0.056
SCR	C	1386	1314	1237
	E	1469	1379	1297

This appendix reports the detailed results of one single replication in the simulation study regarding balanced speed-accuracy effects. In this specific replication, average PE was set to 0.20 and β_j (the SAT effect size) was 20 in RT and 0.05 in PE. The means for all seven measures in the study are shown per cell of the 2 (Test: control vs. experimental) by 3 (SAT Settings: speed, neutral or accuracy) repeated measures design in Table B1. This table shows that in the speeded conditions RTs are about 20 ms faster than in the neutral condition in both the control and the experimental condition. Similarly, RTs are about 20 ms slower in the
